# Linking prey biomass and Neanderthal population in a challenging upland landscape in the Late Pleistocene: the upper valley of the Lozoya River (Spain)

**DOI:** 10.1007/s10980-026-02350-x

**Published:** 2026-04-21

**Authors:** Beatriz Trejo, Marco Vidal-Cordasco, Ana B. Marín-Arroyo, Enrique Baquedano, Juan Luis Arsuaga, Theodoros Karampaglidis, Guillermo Rodríguez-Gómez

**Affiliations:** 1 Departamento de Geodinámica, Estratigrafía y Paleontología, Universidad Complutense de Madrid, C/ José Antonio Novais 12, 28040 Madrid, Spain; 2 Centro UCM-ISCIII de Evolución y Comportamiento Humanos, Avd/ Monforte de Lemos5, Pabellón 14, 28029 Madrid, Spain; 3 Grupo de I+D+I EvoAdapta (Evolución Humana y Adaptaciones Durante la Prehistoria), Departamento Ciencias Históricas, Universidad de Cantabria, Santander, Spain; 4 Department of Zoology, University of Cambridge, Cambridge, UK; 5 Museo Arqueológico y Paleontológico de La Comunidad de Madrid, Plaza de Las Bernardas S/N, 28801 Alcalá de Henares, Spain; 6 Institute of Evolution in Africa, Calle de Covarrubias 36, 28010 Madrid, Spain; 7 Departamento de Ingeniería Geológica y Minera, Universidad de Castilla-La Mancha, Toledo, Spain; 8 Department of Archaeology, Hebrew University of Jerusalem, Jerusalem, Israel; 9 Departamento de Zoología, Universidad de Sevilla, Sevilla, Spain

**Keywords:** Middle Paleolithic, Species distribution models, Paleoecology, Iberian Peninsula, Large herbivorous mammal, Herbivore biomass

## Abstract

*Context* The mountainous and seasonally variable landscapes of the Late Pleistocene period posed major challenges for hunter-gatherers, shaping their ecological strategies and demographic limits. In the central Iberian Peninsula, the landscape played a decisive role in shaping herbivore communities and human settlement patterns. While the Iberian Peninsula has a rich Paleolithic record, MIS 5 archaeological sites are scarce in its central region, which includes two plateaus and the Sistema Central mountain range. This area likely had unfavorable ecological conditions for human habitation during parts of the Pleistocene, so people were confined to valleys and localized ecological refuges. In this context, the upper valley of the Lozoya River in Madrid, Spain, is home to the Calvero de la Higuera sites, which provide evidence of Neanderthal occupation in a sub-Mediterranean highland environment; *Objetives* This study assesses how such a challenging landscape could sustain local Neanderthal populations through large prey resources; *Methods* Using species distribution models (SDMs) based on fossil occurrences and paleoclimatic data, we estimated the habitat favorability, population density, and biomass of nine large herbivorous mammals recorded at Cueva del Camino (Layer 05, ~ 90 ka); *Results* The SDMs revealed significant differences in habitat favorability among species, with *Cervus elaphus* exhibiting the greatest favorability and *Rupicapra pyrenaica* the least. Incorporating habitat favorability into biomass estimates significantly reduced the potential presence of prey, highlighting the spatial limitations of upland ecosystems. Comparisons with modern ecosystems (e.g., Serengeti, Kruger and Hwange) suggest that the results are ecologically feasible; *Conclusion* Our estimates suggest that the upper valley of the Lozoya River had the potential ecological capacity to support between 14 and 33 Neanderthal individuals. This highlights the delicate ecological balance that may have enabled recurrent Neanderthal presence in the challenging upland environments of central Iberia.

**Supplementary Information:**

The online version contains supplementary material available at 10.1007/s10980-026-02350-x.

## Introduction

**Fig. 1:**
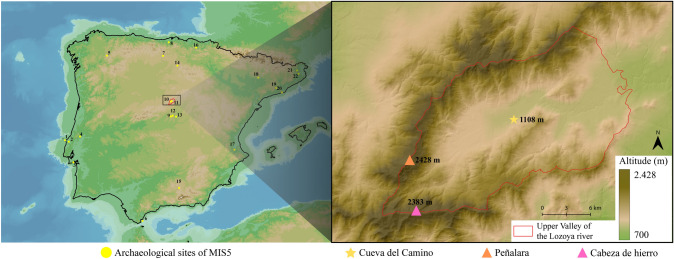
Location of the upper Lozoya River valley and the Cueva del Camino site on the Iberian Peninsula, along with other MIS5 sites. 1: Furninha; 2: Gruta Nova da Columbeira; 3: Figueira Brava Cave; 4: Gruta da Oliveira; 5: Cova Eiros; 6: Vanguard Cave; 7: Cueva Corazón; 8: El Castillo; 9: Arriaga; 10: Cueva del Camino; 11: Buena Pinta; 12: Estanque de Tormentas de Butarque; 13: Preresa; 14: Galería de las Estatuas; 15: Carihuela; 16: Artazu; 19: Cova Negra; 20: Estret de Tragó; 21: Can Costella; 22: Cova del Rinoceront; 23: Arbreda; 24: Can Garriga (modified from Molino et al. 2025)

In the central Iberian Peninsula, the landscape itself played a decisive role in shaping both the composition of herbivore communities and the patterns of human occupation. Although the Iberian Peninsula is one of the key regions for the study of the European Palaeolithic record due to its rich archaeological and paleontological heritage, the central region shows a limited representation of MIS 5 archaeological sites (see Molino et al. 2025; Fig. 1A), which provides the regional context for Cueva del Camino. This central area, encompassing two plateaus and the Sistema Central mountain range, presented unfavorable ecological conditions for human habitation in different periods of the Paleolithic (Burke et al. 2017; Wolf et al. 2018; Sala et al. 2020). Consequently, human occupation may have been discontinuous and constrained by environmental limitations. However, certain valleys and ecological enclaves, i.e., geographically restricted environments with locally buffered conditions and concentrated resources that increase habitat favorability and potential prey biomass, could have provided more favorable conditions for habitation and resource exploitation (Buitrago Villaplana 1993).

This study focuses on the herbivorous mammal community present at the Cueva del Camino site (40°55′ N, 3°48′ W at 1,108 m asl), which belongs to the Calvero de la Higuera archaeological complex (Pinilla del Valle, Madrid, Spain), located in the surroundings of the upper valley of the Lozoya River in the Sierra de Guadarrama (Madrid, Spain) (Fig. 1).

The Layer 05 of this site has a chronology of 90.9 ± 7.8 ka and 91.6 ± 8.1 by thermoluminescence in sediment samples (Pérez-González et al. 2010) and contains one of the richest mammal assemblages of the Iberian Peninsula during the Pleistocene (Arsuaga et al. 2011). The origin of this accumulation is associated with its use by the spotted hyena (*Crocuta crocuta*) as a den or lair (Díez 1993; Arsuaga et al. 2010). Among the species represented, there is a significant presence of ungulates, especially fallow deer (*Dama dama*), as well as carnivores and Neanderthals (Álvarez-Lao et al. 2013). Human remains found include an upper right M1 and an upper right M3, as well as more than 52 lithic remains from campaigns conducted from 2002 to 2009 (Alférez et al. 1982; Alférez and Roldán 1992; Gómez-Robles et al. 2007; Martinón-Torres et al. 2007, 2008; Arsuaga et al. 2012).

Cueva del Camino offers an exceptional opportunity to explore the relationship between Neanderthal populations and the mountainous landscapes of central Iberia. Its faunal diversity and evidence of Neanderthal presence make it a key site for evaluating the ecological potential of the upper valley of the Lozoya River and understanding how these human groups adapted to high-altitude environments during MIS 5 (Buitrago Villaplana 1993; Álvarez-Lao et al. 2013; Molino et al. 2025). Recent studies of Late Pleistocene mountainous regions suggest that rugged highland landscapes constrained human demography and subsistence, promoting sparse and ephemeral occupation by small, highly mobile groups that ranged widely, relied on temporary camps, and adapted hunting strategies to fluctuating climatic and ecological conditions (Malinsky-Buller et al. 2021; Dewar et al. 2024; Yeshurun 2025). In this broader context, the Lozoya uplands may illustrate how high prey biomass could have made a rugged setting more attractive, potentially leading to repeated Neanderthal presence in the valley, though not necessarily continuous (Molino et al. 2025). Given the evidence for repeated human presence in the upper valley of the Lozoya River, this area stands out as a focal setting for human activity, offering valuable insights into the interplay between humans and their environments from the Middle Pleistocene to the Holocene (Baquedano et al. 2010, 2014; Arsuaga et al. 2012; Sánchez-Romero et al. 2017; Moclán et al. 2018, 2021). In order to understand the ecological conditions that made this possible, it is necessary to evaluate the influence of the regional environment on the distribution and abundance of large herbivores, which were the primary source of food and biomass for human groups during the Pleistocene (Roebroeks 2001; Owen-Smith and Mills 2008; Rodríguez-Gómez et al. 2014).

Reconstructing the distribution and abundance of species in the past requires linking their ecological preferences with the environmental characteristics of their landscapes. In this context, herbivore communities play an important role in population dynamics, as well as in the structure and functioning of the ecosystems, making them highly significant for their reconstruction (Grange and Duncan 2006). These communities are not only influenced by climatic parameters, such as temperature and precipitation, but are also affected by other local factors such as topography, the distribution of water bodies, natural barriers, snow cover, among others (Guthrie 1990; Discamps et al. 2011, 2014). This makes them highly relevant parameters in the ecosystems that species inhabit, with their inclusion being essential for developing distribution models.

Species Distribution Models (SDMs) are a heterogeneous group of techniques, with statistical and/or mechanistic approaches, used to determine the distribution area and predict species’ presence across space and/or time, relating their known occurrences with the environmental conditions in these locations (Guisan and Zimmermann 2000; Guisan and Thuiller 2005; Elith and Leathwick 2009; Svenning et al. 2011; Varela et al. 2011). The use of these techniques is becoming increasingly important in paleobiology, as it helps to understand the evolution of biodiversity and its geographic patterns (Lomolino et al. 2010; Svenning et al. 2011), providing quantitative predictions with potentially high resolution of past organism distributions, a statistical evaluation of the drivers of these distributions, as well as information on species and community dynamics (Svenning et al. 2011).

In this context, the application of SDMs provides the basis for the present study, which aims to reconstruct the environment inhabited by Neanderthals during the Late Pleistocene in the upper valley of the Lozoya River when the sediments of the Layer 05 of Cueva del Camino were deposited. Based on the herbivore community represented at the site, the study further estimates the animal resources that these humans could have exploited. To achieve this, we have four specific objectives: (1) to create a potential spatial distribution model of the suitable habitats for the herbivorous mammal species present in the upper valley of the Lozoya River environment; (2) to estimate the population densities and the expected number of individuals for each species; (3) to calculate the carrying capacity of the ecosystem based on the population densities of prey; (4) and to infer the Neanderthal population from the carrying capacity. All estimates are interpreted at an annual scale as time-averaged baselines for the 80–100 ka window, which was selected to match the chronology of Cueva del Camino and its associated uncertainty.

## Materials and methods

The study focuses on the upper valley of the Lozoya River, which covers 434.58 km^2^ (see Karampaglidis et al. 2015) (Fig. 1). The upper valley of the Lozoya River is a structurally controlled intramontane basin developed within a NE–SW-oriented tectonic depression (a pop-down structure) of the Spanish Central System (De Vicente et al. 2007). The valley floor exhibits a broad, relatively open morphology, characterized by braided to wandering gravel-bed channels linked to high sediment supply (Karampaglidis et al. 2015). Glacial and periglacial landforms are confined to the highest elevations (∼ 1600–2400 m) (Pedraza and Carrasco 2006; Carrasco et al. 2022), where cirques and morainic deposits provide clear evidence of Late Pleistocene glaciation. In addition, slope processes (colluvial and gravitational deposits), karst features developed in carbonate lithologies, and widespread weathering forms contribute to the geomorphic complexity of the landscape. Overall, the upper valley of the Lozoya River constitutes a polygenetic geomorphic system in which structural control, lithology, fluvial incision, and Quaternary climatic variability interact to produce a stepped, multi-level valley morphology (Karampaglidis et al. 2015). At 1108 m a.s.l., Cueva del Camino lies within a mountainous basin bordered to the north by the Montes Carpetanos, where Peñalara reaches 2428 m, and to the south by the Cuerda Larga range, where Cabeza de Hierro reaches 2383 m. On this basis, we treat the upper valley of the Lozoya River as a geomorphologically coherent unit and, for analytical purposes, as a semi-closed system with only one possible exit in the northeastern sector. In this sense, the upper valley of the Lozoya River constitutes a relatively small and geomorphologically coherent study area, allowing the model outputs to be interpreted at a local landscape scale. Although glacial, fluvial, and karst processes have acted on the valley since Cueva del Camino was formed, the available evidence indicates that the valley’s morphology (e.g., confinement, steep altitudinal gradients, and broad landform configuration) has remained largely stable. Therefore, in this study, we use the current morphology of the valley, while acknowledging that minor geomorphological changes may have occurred since the formation of Cueva del Camino (Karampaglidis et al. 2015).

### Species occurrence data

We modelled the potential distribution of the main herbivorous mammals recorded at the Cueva del Camino site with body mass > 10 kg, as these taxa constitute the core prey base potentially available to hominins and the trophic network in which they would be integrated (e.g., Rodríguez-Gómez et al. 2013, 2014, 2016, 2017a, 2017b). The selected species were: *Stephanorhinus hemitoechus* (steppe rhinoceros), * Equus ferus* (horse), *Bos primigenius* (aurochs), *Rupicapra pyrenaica* (chamois), *Cervus elaphus* (red deer), *Dama dama* (fallow deer), *Capreolus capreolus* (roe deer), *Sus scrofa* (wild boar), and *Castor fiber* (European beaver) (Arsuaga et al. 2012; Álvarez-Lao et al. 2013; Baquedano et al. 2014; Molino et al. 2025).

**Fig. 2:**
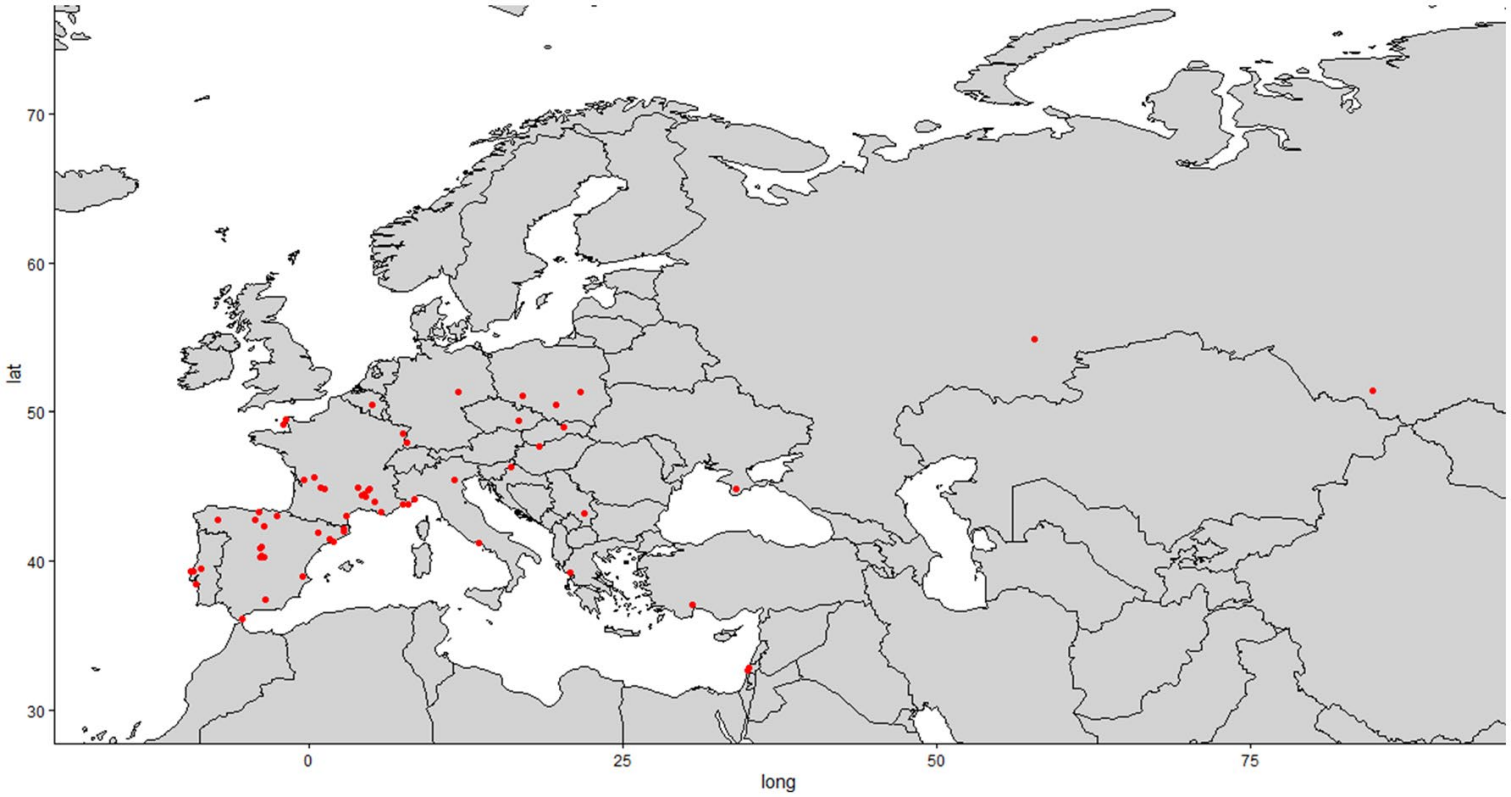
Location of archaeological and paleontological sites containing remains of the selected species found at the Cueva del Camino site (see Supplementary Material)

We then compiled fossil occurrences of these taxa across Eurasia from published sources and available databases (see Supplementary Material) (Fig. 2). For our analyses, we retained an occurrence only when it could be assigned to a specific site and stratigraphic unit, geographic coordinates were available (or could be reliably georeferenced from published site information), and the associated age estimate fell within 80–100 ka, encompassing the chronology of Cueva del Camino and its reported uncertainty (see above). The resulting number of points is presented in Table 1 as the "original dataset". Although fossil occurrences were compiled across a broad geographical area for model calibration, the palaeoecological interpretation of the results is explicitly restricted to the upper valley of the Lozoya River as a local study area.

**Table 1:** Number of observations for each species

	Original dataset	Filtered dataset
*Stephanorhinus hemitoechus*	7	7
* Equus ferus*	47	41
*Bos primigenius*	69	50
*Rupicapra pyrenaica*	39	30
*Cervus elaphus*	138	111
*Dama dama*	50	38
*Capreolus capreolus*	77	64
*Sus scrofa*	78	61
*Castor fiber*	45	36

Original dataset is the number of observations collected and the filtered dataset is the result number of observations after keeping only one occurrence per time slice and grid cell

Spatial and temporal biases in occurrence data are well-documented issues affecting model predictions. However, environmental filtering of occurrence records has been shown to improve model accuracy by reducing redundancy in environmental hyperspace (Varela et al. 2014). To address this, we filtered the original dataset by retaining only one occurrence per pixel and time step (1 ka) for each species, leading to the number of points presented in Table 1 as the "filtered dataset".

### Climatic predictors

The climatic variables were obtained from the R package pastclim (Leonardi et al. 2023) using the dataset of Krapp et al. (2021). These variables, initially at a resolution of 0.5°, were downscaled to 30 s using bilinear and weighted interpolation, taking the spatial resolution of the WorldClim (Fick and Hijmans 2017) current climate dataset as a reference. As a result, eighteen of the twenty-two bioclimatic variables were generated, excluding NPP, rugosity, and biomes, as they do not appear in the WorldClim variables (Table 2). We also included a variable for biomes, calculated from the classification of Holdridge (1947) using temperature and precipitation variables.

**Table 2:** Climatic variables used for distribution modelling

Variable name	Description
bio01	Annual mean temperature
bio04	Temperature seasonality
bio05	Maximum temperature of warmest month
bio06	Minimum temperature of coldest month
bio07	Temperature annual range (bio05-bio06)
bio08	Mean temperature of wettest quarter
bio09	Mean temperature of driest quarter
bio10	Mean temperature of warmest quarter
bio11	Mean temperature of coldest quarter
bio12	Annual precipitation
bio13	Precipitation of wettest month
bio14	Precipitation of driest month
bio15	Precipitation seasonality (coefficient of variation)
bio16	Precipitation of wettest quarter
bio17	Precipitation of driest quarter
bio18	Precipitation of warmest quarter
bio19	Precipitation of coldest quarter
altitude	Altitude over the sea level
biome	Biome type

### Potential distribution modelling

To model species distributions, we used an ensemble forecasting approach incorporating four modeling techniques: (1) Generalized Linear Models (GLMs), (2) Generalized Additive Models (GAMs), (3) Maximum Entropy (Maxent), and (4) Bayesian Additive Regression Trees (BART). These SDMs require occurrence data, a set of climatic predictors, and absence/pseudoabsence data. To maximize the ecological information available, we combined occurrence and climatic data across all time intervals in which each species was present (see Nogués-Bravo 2009; Maiorano et al. 2013). This approach enhances niche characterization by incorporating the widest possible range of environmental conditions while minimizing the effects of environmental truncation on niche estimation (Guisan et al. 2014).

For each species, we generated pseudo-absences following a density-dependent approach, ensuring a higher concentration of pseudoabsences in areas where occurrences were denser (Phillips and Dudík 2008; Syfert et al. 2013; Roy‐Dufresne et al. 2019). This method helps prevent pseudoabsences from being placed in regions where fossil records are absent due to low fossilization potential rather than the true absence of the species (Fourcade et al. 2018; Title and Bemmels 2018; Guevara et al. 2018). Pseudoabsences were generated for each species and time period within a defined calibration area. This area was determined by creating a buffer enclosing all fossil localities where the species was present, with a radius equal to 10% of the maximum distance between fossil occurrences. Within this area, pseudo-absences were randomly sampled from the corresponding climatic layers for each time slice, maintaining a ratio of 50 pseudo-absences per presence point.

Since the number of predictor variables influences the complexity of the models—where both an excessive number of variables and high collinearity can affect the results (Phillips et al. 2006; Li et al. 2020)—we checked for correlated variables, selecting those with an r^2^ < 0.8 to avoid redundancy, resulting in the exclusion of “bio06”, “bio07”, “bio10”, “bio11”, “bio13”, “bio16”, “bio17” and “biome”.

SDMs estimate habitat suitability or presence probability, as well as habitat favorability, a prevalence-independent measure of how favorable local environmental conditions are for a species. Among these metrics, suitability and presence probability can be influenced by species prevalence, which can hinder direct cross-species comparisons. In contrast, favorability is independent of prevalence, enabling direct comparison across species and models. In practical terms, favorability expresses the degree to which a given location matches the environmental conditions associated with the species’ occurrences, with higher values indicating more favourable conditions. In this study, we used favorability to compare potential spatial distributions among species.

Model performance was assessed using the area under the curve (AUC) of the receiver operating characteristic (ROC) curve (Swets 1988). The ROC curve represents the relationship between the true positive rate (i.e., the proportion of correctly predicted presences among all observed presences) and the false positive rate (i.e., the proportion of incorrectly predicted presences among all observed absences). The AUC value provides a single measure of overall accuracy and is widely used as a standard metric for model evaluation (DeLeo 1993; Fielding and Bell 1997; Li et al. 2020). It is also a common evaluation criterion in species distribution modeling (Fielding and Bell 1997; Phillips et al. 2006; Beale and Lennon 2012; Daszak et al. 2013; Li et al. 2020) and is not sensitive to species prevalence (Hanley and McNeil 1982; Li et al. 2020).

Each modeling technique was repeated using all possible variable combinations, selecting the model with the highest AUC. Typically, AUC values range from 0.5 to 1, with random models having a value of 0.5 and perfect models reaching a value of 1; thus, the closer the value is to 1, the better the model prediction (Phillips and Dudík 2008; Li et al. 2020). In our case, only SDMs with an internal AUC value greater than 0.7 (Swets 1988) were included in the ensemble. Finally, for each species, the ensemble SDMs were projected every 1000 years for the period between 80 and 100 ka, using time-averaged environmental predictors. Although seasonal dynamics are expected in mid- and high-altitude environments, our modeling framework is designed to estimate annual resource availability. Therefore, the resulting model outputs and the derived density and biomass estimates should be interpreted as annual-average baselines of potential distribution and carrying capacity across the upper valley of the Lozoya River.

### Carrying capacity and the relation between prey and predator biomass

Numerous models link the carrying capacity of different ecosystems with Net Primary Production (NPP), based on empirical evidence of the relationship between NPP and herbivore abundance (Coe et al. 1976; McNaughton et al. 1991; Santini et al. 2018; Vidal-Cordasco et al. 2022, 2023). The carrying capacity of an ecosystem is considered the maximum theoretical population size that a particular environment can sustain over a given period of time (see Sayre 2008). In this study, we will consider the carrying capacity of the Cueva del Camino ecosystem as the sum of the biomasses of herbivorous large mammal populations that the ecosystem could support, as proposed by Coe et al. (1976). To estimate the carrying capacity of the Cueva del Camino paleoecosystem, we first obtained NPP values for the period between 100,000 and 80,000 years ago, which covers both the Cueva del Camino chronology and the associated error range. For this, we used two empirical models—the Miami model (Lieth 1975) and the NCEAS model (Del Grosso et al. 2008)—to estimate NPP values for the study area at 1 ka intervals. Both models use mean annual temperature and annual precipitation as predictors and apply the law of the minimum, assuming that primary productivity is limited by the single climatic factor that yields the lowest NPP value. However, unlike the Miami model, the NCEAS model accounts for differences between biome types, distinguishing between tree-dominated and non-tree-dominated ecosystems. In this study, we used the NPP estimations for tree-dominated ecosystems, as these values were higher than those for non-tree-dominated ecosystems, taking these values as the maximum potential NPP for our study area. As a result, we obtained two sets of NPP values—one from the Miami model and one from the NCEAS model—which were used separately in the following estimations.

Subsequently, the total herbivore biomass (THB) that the ecosystem could support was calculated using the predictive equation proposed by Vidal-Cordasco et al. (2022, 2023):

1 $$\log_{10} \left[ {THB} \right] \, = \, 1.401 \, \times \, \log_{10} \left[ {NPP} \right] \, - \, 0.642,$$

where the values of THB and NPP are expressed in g m^−2^ year^−1^.

It is assumed that herbivore abundance depends on the food web regulation process for a given NPP, the allometric relationship between body mass and population density, and the structure and composition of herbivore communities (Vidal-Cordasco et al. 2023). Therefore, the potential population densities (*D*_*P*_) for each species were obtained from the THB values. This was done using the equation proposed by Vidal-Cordasco et al. (2022):

2 $$D_{{P_{i} }} = \frac{THB}{{\mathop \sum \nolimits_{i = 1}^{n} W_{{P_{i} }}^{ 0.25} }} \times W_{{P_{i} }}^{ - 0.75} ,$$

where ${D}_{{P}_{i}}$ is the potential population density of species i expressed in individuals/km^2^, and ${W}_{{P}_{i}}$ is the mean body mass of the species i population in kg, as proposed by Molino et al. (2025).

Using these *D*_*P*_ values, the expected density (*D*_*E*_) for each species was obtained by multiplying *D*_*P*_ by the favorability values from the SDMs. In this context, favorability is used to downscale potential densities to expected densities under average annual conditions across the valley. This approach assumes that the potential density (*D*_*P*_) represents the maximum density a species could reach under optimal environmental conditions and ensures that *D*_*E*_ is proportionally related to the favorability of the ecosystem in which the species occurs. By scaling *D*_*P*_ according to favorability, we account for the fact that less suitable habitats are likely to support lower population densities. This assumption aligns with ecological patterns described by Channell and Lomolino (2000), who showed that species tend to occur at lower and more variable densities as environmental conditions become less favorable, particularly toward the periphery of their ranges. Likewise, we observed that in modern ecosystems there is a reduction in observed biomass compared to the potential biomass that the ecosystem could support (see Supplementary Material). Given that our study area covers 434.58 km^2^, we estimated the total number of individuals (*I*_*E*_) for each species accordingly. Finally, the carrying capacity of the upper valley of the Lozoya River was determined by calculating the prey biomass (*PB*_*E*_):

3 $$PB_{E} = \mathop \sum \limits_{i = 1}^{n} \left( {D_{{E_{i} }} \times W_{{P_{i} }} } \right),$$

where ${{D}_{E}}_{\mathrm{i}}$ is the expected population density of species i expressed in individuals/km^2^ and ${W}_{{P}_{i}}$ is the average body mass of the population of species i expressed in kg, as obtained by Molino et al. (2025).

The predator biomass value was also obtained, as both prey and predator biomass help to describe the relative changes in the shape of the biomass trophic pyramid, which illustrates how total biomass is distributed among communities at different trophic levels of the food web (Elton 1927; Odum 1971; Trebilco et al. 2013; Hatton et al. 2015). This was calculated using the equation by Hatton et al. (2015) for a savanna ecosystem:

4 $$DB_{E} = 0.094 \times PB_{E}^{0.73} ,$$

where *DB*_*E*_ is the expected predator biomass and *PB*_*E*_ is the expected prey biomass, obtained from Eq. (3). These values are expressed in kg/km^2^/year.

To validate this methodology, we first compared the current NPP values obtained from the Miami and NCEAS models (using temperature and precipitation variables from WorldClim (Fick and Hijmans 2017)) with those reported by the EEA (European Environment Agency 2023) for the year 2000 in the upper valley of the Lozoya River. This allowed us to determine which model best approximates the expected values in our study area (see Supplementary Material). Subsequently, we compared the biomass reduction values (i.e., the proportion of potential biomass not realized in the observed biomass) observed in our study area with those reported by Hatton et al. (2015) for three ecosystems (Africa, India, and North America) and by Rodríguez et al. (2014) for ecosystems in different parts of the world (Global) (see Supplementary Material).

## Results

### Species potential distribution

#### Model performance and variable importance

The species distribution models (SDMs) showed varying performance depending on the method used, as can be seen from the AUC values obtained (Table 3). The techniques that yielded the best performance were BART, GAM and Maxent, which consistently achieved higher AUC values (always above 0.8) for all species, except for *Stephanorhinus hemitoechus*, which had a value of 0.527 for Maxent. In contrast, GLM exhibited the lowest AUC values, often around 0.7.

**Table 3:** Model performance and variable importance for each technique (GLM, GAM, Maxent, and BART) and species

Species	SDM Method	AUC	Predictive variables										
			bio01	bio04	bio05	bio08	bio09	bio12	bio14	bio15	bio18	bio19	altitude
*Stephanorhinus hemitoechus*	GLM	0.727	–	0.823	–	0.458	1.127	0.819	–	0.283	–	–	–
	GAM	0.981	–	0.703	–	0.508	0.422	1.419	–	–	–	–	1.173
	Maxent	0.527	–	–	–	0	100	–	–	–	–	–	–
	BART	0.905	0.245	0.260	–	–	0.249	0.246	–	–	–	–	–
*Equus ferus*	GLM	0.747	–	3.871	2.853	0.434	–	–	–	–	–	–	1.296
	GAM	0.891	0.554	–	1.427	1.336	–	–	–	–	1.949	1.139	3.529
	Maxent	0.864	45.28	–	–	10.54	–	3.07	–	–	–	–	41.11
	BART	0.907	0.326	–	–	–	–	–	–	–	–	0.333	0.341
*Bos primigenius*	GLM	0.700	1.006	0.274	–	1.771	–	–	2.761	2.562	–	2.336	3.392
	GAM	0.869	1.434	0.881	–	–	–	2.248	–	3.675	–	–	4.024
	Maxent	0.840	26.22	0.54	–	–	–	30.47	–	11.61	–	–	31.16
	BART	0.914	–	–	–	–	–	0.246	0.238	0.246	–	–	0.269
*Rupicapra pyrenaica*	GLM	0.661	1.650	1.455	–	–	0.660	–	–	0.567	0.176	0.250	1.165
	GAM	0.931	0.804	0.950	–	–	2.921	–	–	3.851	1.865	1.662	5.223
	Maxent	0.880	2.54	3.89	–	0.01	–	5.19	–	2.44	–	–	85.92
	BART	0.906	–	–	–	–	–	–	–	0.322	–	0.332	0.346
*Cervus elaphus*	GLM	0.834	0.274	4.303	–	–	–	2.501	–	3.949	3.681	–	1.619
	GAM	0.918	6.493	0.324	–	1.377	–	4.877	–	–	3.184	–	4.471
	Maxent	0.916	27.17	–	7.39	5.50	–	44.43	–	1.54	–	–	13.97
	BART	0.938	0.325	–	–	–	–	0.331	–	–	–	–	0.344
*Dama dama*	GLM	0.735	3.046	2.045	–	–	2.391	–	1.772	2.001	–	–	4.062
	GAM	0.914	–	0.754	1.746	–	–	2.794	–	2.817	–	2.940	3.343
	Maxent	0.872	24.09	14.30	–	0.76	–	10.89	–	15.24	–	–	34.72
	BART	0.920	–	–	–	–	–	–	0.322	0.319	–	–	0.359
*Capreolus capreolus*	GLM	0.708	1.547	–	–	0.990	–	–	3.414	4.581	–	2.445	3.374
	GAM	0.880	4.967	1.335	–	1.805	–	–	3.883	1.358	–	2.702	5.757
	Maxent	0.850	36.71	2.58	–	0.86	–	36.30	–	5.90	–	–	17.65
	BART	0.913	0.321	–	–	–	–	0.327	–	–	–	–	0.352
*Sus scrofa*	GLM	0.708	1.079	0.674	–	2.362	–	–	3.042	3.378	–	3.880	2.794
	GAM	0.891	4.655	3.600	–	2.263	–	3.900	–	3.523	–	–	4.262
	Maxent	0.866	28.25	4.22	–	1.62	–	38.95	–	4.36	–	–	22.60
	BART	0.936	0.310	–	–	–	–	0.336	–	–	–	–	0.355
*Castor fiber*	GLM	0.781	3.393	–	1.836	2.679	–	3.016	–	2.380	–	–	4.108
	GAM	0.927	0.636	–	0.383	–	–	0.760	–	3.308	1.746	–	4.865
	Maxent	0.907	9.20	–	0.17	1.50	–	37.21	–	14.18	–	–	37.73
	BART	0.928	0.333	–	0.330	–	–	–	–	–	–	–	0.336

*GLM* Generalized Linear Model, *GAM* Generalized Additive Model, *Maxent* Maximum Entropy, *BART* Bayesian Additive Regression Trees

**Fig. 3:**
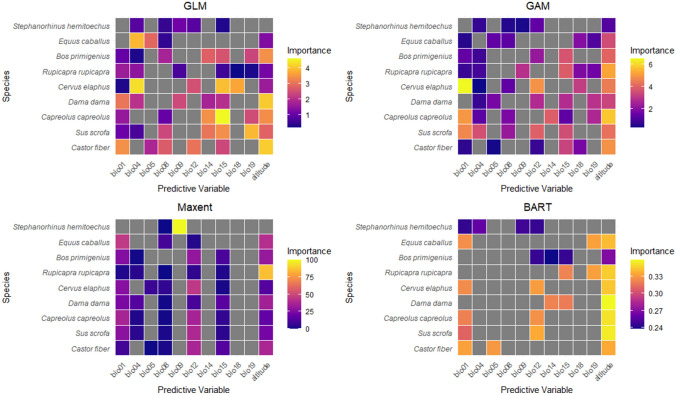
Heatmap of variable importance for each technique (GLM, GAM, Maxent, and BART) and species. GLM = Generalized Linear Model; GAM = Generalized Additive Model; Maxent = Maximum Entropy; BART = Bayesian Additive Regression Trees

Differences in the frequency of variable selection were observed across models (Fig. 3). The variables “bio01”, “bio08”, “bio12” and “altitude” were the most frequently selected, being present in at least one SDM technique for every species (Fig. 3). Temperature and precipitation seasonality (“bio04” and “bio15”, respectively) were also frequently selected, with only one case for each variable (*Castor fiber* and * Equus ferus*, respectively) where they were not included (Fig. 3). On the other hand, the least frequent variable in the SDMs was “bio09”, being selected for only three species: *Stephanorhinus hemitoechus*, *Rupicapra pyrenaica* and *Dama dama*. The selection of variables also varied between models, with GLM and GAM tending to use more variables than Maxent and BART, and the latter tends to use “altitude” more often and give more importance (Fig. 3).

Regarding variable importance, the way it is expressed differs across SDM techniques due to the distinct criteria each method uses. In GLM and GAM, it is represented by the absolute value of the t-statistic, reflecting the strength of the relationship between the predictor and the response variable. Maxent quantifies it as the percentage contribution of each variable to the overall model performance, while BART defines it based on the proportion of decision tree branches in which a variable appears.

Variation in variable importance was found across models and species. “Altitude” was the most relevant variable overall, being important for every species, followed by “bio01”, which was relevant for five species (Fig. 3). The least important variables were “bio05”, “bio08”, “bio14” and “bio18”, as they were not of relevance to any species (Fig. 3).

#### Analysis of the potential distribution

The species distribution models (SDMs) generated for the nine species analyzed in the upper valley of the Lozoya River reveal differences in habitat favorability, reflected in the spatial variability of the predicted values. For some species, the ecosystem exhibits better favorability, such as *Cervus elaphus* and *Capreolus capreolus*, with mean favorability values exceeding 0.6, while others show less favorability, such as *Stephanorhinus hemitoechus*, * Equus ferus*, *Bos primigenius* and *Castor fiber*, with values below 0.5 (Table 4). In contrast, the ecosystem does not appear to be suitable for *Rupicapra pyrenaica*, which has a mean favorability of 0.067 (Table 4).

**Table 4:** Mean habitat favorability and standard deviation (SD) for each species

	Mean favorability	SD
*Stephanorhinus hemitoechus*	0.402	0.036
* Equus ferus*	0.346	0.036
*Bos primigenius*	0.429	0.015
*Rupicapra pyrenaica*	0.067	0.010
*Cervus elaphus*	0.642	0.012
*Dama dama*	0.589	0.009
*Capreolus capreolus*	0.622	0.018
*Sus scrofa*	0.580	0.030
*Castor fiber*	0.310	0.043

**Fig. 4:**
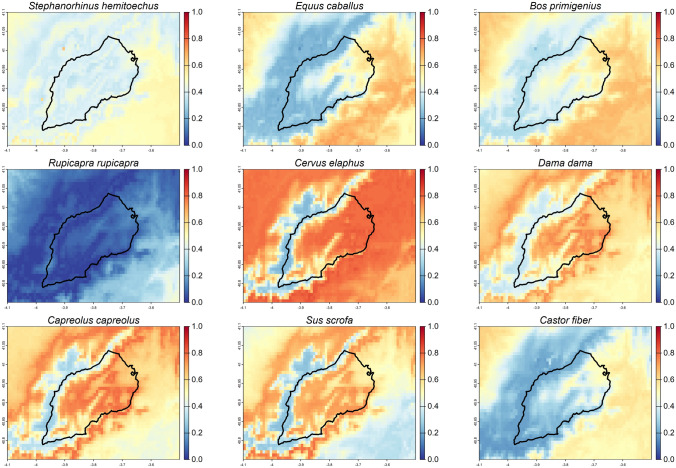
Mean Species Distribution Models (SDMs) for each species in the upper valley of the Lozoya River (delimited area) and its surroundings

*Dama dama*, *Capreolus capreolus* and *Sus scrofa* show a slight preference for the mid-altitude area, whereas * Equus ferus*, *Bos primigenius*, *Cervus elaphus* and *Castor fiber* prefer low-altitude zones. However, all species avoid areas of higher elevation to varying degrees. Some species, such as *Cervus elaphus*, exhibit broadly distributed habitat suitability across the study area, while others, like *Stephanorhinus hemitoechus* and *Rupicapra pyrenaica*, show low favorability throughout most of the region (Fig. 4).

### Population densities

As shown in Table 5, the potential population density (*D*_*P*_) values, the expected population density values (*D*_*E*_), and the expected number of individuals in the study area (*I*_*E*_) were obtained. The NPP values were 1091.53 g/m^2^/year for the Miami model and 504.18 g/m^2^/year for the NCEAS model.

**Table 5:** Potential population densities in individuals/km^2^ (D_P_), expected population densities in individuals/km^2^ (D_E_) and the expected number of individuals (I_E_) in the upper valley of the Lozoya River, for each NPP model (mean NPP = 1091.53 (sd = 14.34) g/m^2^/year for Miami model and mean NPP = 504.18 (sd = 8.35) g/m^2^/year for NCEAS model)

	Miami model			NCEAS model		
	D_P_	D_E_	I_E_	D_P_	D_E_	I_E_
*Stephanorhinus hemitoechus*	0.78	0.32	139	0.27	0.11	48
* Equus ferus*	1.93	0.66	287	0.66	0.21	91
*Bos primigenius*	1.46	0.62	269	0.50	0.21	91
*Rupicapra pyrenaica*	13.76	0.89	387	4.69	0.29	126
*Cervus elaphus*	3.62	2.34	1017	1.23	0.76	330
*Dama dama*	5.39	3.11	1352	1.83	1.02	443
*Capreolus capreolus*	12.48	7.89	3429	4.25	2.58	1121
*Sus scrofa*	6.99	4.14	1799	2.38	1.36	591
*Castor fiber*	20.83	6.46	2807	7.09	2.08	904

**Fig. 5:**
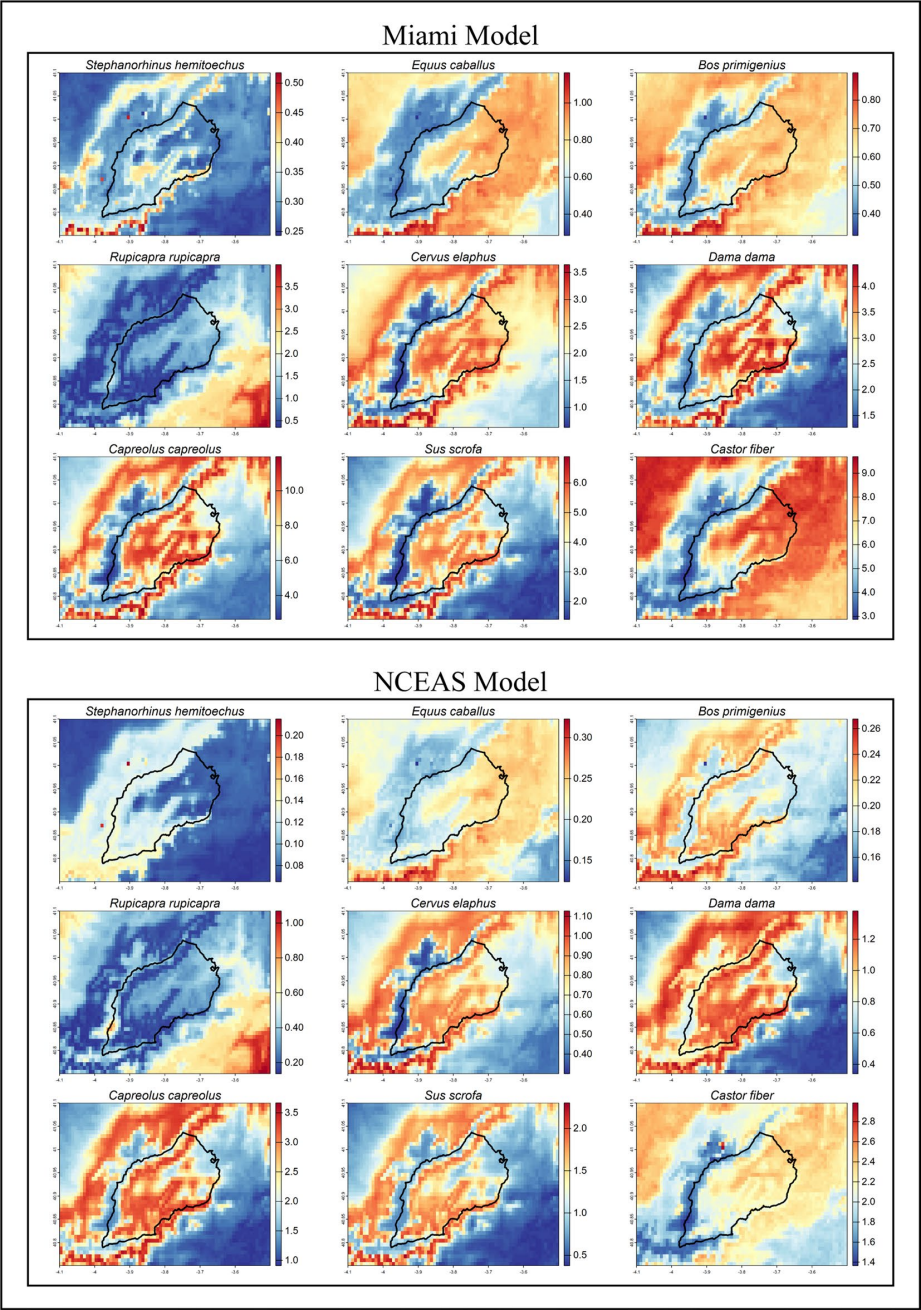
Expected population densities in individuals/km^2^ (D_E_) in the upper valley of the Lozoya River, for each NPP model and species

Differences were found between the population densities obtained from the Miami model and the NCEAS model. The Miami model resulted in higher NPP values, which translated into greater population densities for all species compared to those obtained using the NPP from the NCEAS model (Table 5). Additionally, we found higher interspecific variability in the population densities obtained from the Miami model compared to those from the NCEAS model. For *D*_*P*_, the variance is significantly higher in the densities obtained from the Miami model (s^2^ = 46.76) than in those from the NCEAS model (s^2^ = 5.42). This pattern remains for *D*_*E*_, where variance is also higher in the Miami model (s^2^ = 7.55) than in the NCEAS model (s^2^ = 0.80). This variability led to species with very high densities in the Miami model, such as *Castor fiber* (*D*_*P*_ = 20.83 individuals/km^2^), while others, such as *Stephanorhinus hemitoechus*, had notably low densities (*D*_*P*_ = 0.78 individuals/km^2^). In contrast, the NCEAS model produced more uniform densities among species, with lower variability. This is also reflected in Fig. 5, where the distribution of expected densities varies depending on the NPP model used. In the case of the Miami model, densities are more widely distributed across the landscape, whereas with the NCEAS model, areas of higher density appear more restricted.

The species with the highest *D*_*P*_ values in both models were *Castor fiber*, *Rupicapra pyrenaica*, and *Capreolus capreolus*, which could be related to their lower body masses (24.98 kg for *Capreolus capreolus*, 21.92 kg for *Rupicapra pyrenaica*, and 12.61 kg for *Castor fiber*) (Table 6). Conversely, *Bos primigenius* and *Stephanorhinus hemitoechus* showed the lowest values, as they were the species with the highest body masses (437.66 kg and 1006.27 kg, respectively) (Table 6).

**Table 6:** Average body mass (kg) of each species obtained from Molino et al. (2025) (${W}_{{P}_{i}}$); potential biomass (kg/km^2^/year) obtained without considering the favorability (*PB*_*P*_) and its percentage of contribution; expected biomass (kg/km^2^/year) obtained considering the favorability of the ecosystem (*PB*_*E*_) and its percentage of contribution; percentage of biomass reduction (%Reduction); total biomass of prey and predators (kg/km^2^/year) from each model; and the prey-predator biomass ratio (kg prey/kg predator) for each model

	${W}_{{P}_{i}}$	Miami model			NCEAS model		
		PB_P_	PB_E_	%Reduction	PB_P_	PB_E_	%Reduction
*Stephanorhinus hemitoechus*	1006.27	785.08 (19%)	318.17 (17%)	59.47	267.34 (19%)	108.49 (18%)	59.42
* Equus ferus*	301.35	580.77 (14%)	199.06 (11%)	65.72	197.77 (14%)	64.11 (11%)	67.58
*Bos primigenius*	437.66	637.56 (15%)	272.35 (15%)	57.28	217.10 (15%)	89.93 (15%)	58.58
*Rupicapra pyrenaica*	21.92	301.61 (7%)	19.52 (1%)	93.53	102.71 (7%)	6.37 (1%)	93.80
*Cervus elaphus*	129.88	470.57 (11%)	304.21 (16%)	35.35	160.24 (11%)	98.17 (16%)	38.74
*Dama dama*	76.51	412.26 (10%)	238.19 (13%)	42.22	140.38 (10%)	78.40 (13%)	44.15
*Capreolus capreolus*	24.98	311.63 (8%)	197.01 (11%)	36.78	106.12 (8%)	64.36 (11%)	39.35
*Sus scrofa*	54.09	378.02 (9%)	224.06 (12%)	40.73	128.73 (9%)	76.08 (12%)	64.36
*Castor fiber*	12.61	262.67 (6%)	81.47 (4%)	68.98	89.45 (6%)	26.28 (4%)	70.62
Prey biomass		4140.17	1854.04	55.22	1409.83	609.57	56.76
Predator biomass		41.01	22.85	44.29	18.61	10.14	45.49
Prey-predator Biomass ratio		100.95	81.15	19.62	75.75	60.09	20.67

However, when habitat favorability was incorporated into the calculation of population density (*D*_*E*_), the resulting values indicated changes in community structure. We observed that the species with the highest *D*_*E*_ values in both models were *Capreolus capreolus*, *Sus scrofa*, and *Castor fiber*, with *Capreolus capreolus* being the species with the highest population density in this case (Table 5).

### Prey and predator biomass

As was observed for population densities, the biomass obtained from the Miami model showed higher values than those from the NCEAS model (Table 6). This difference is also reflected in the total biomass of prey and predators. Using the Miami model, potential prey biomass (*PB*_*P*_) reaches 4140.17 kg/km^2^/year, whereas with the NCEAS model, this value is considerably lower (1409.83 kg/km^2^/year). Similarly, predator biomass in the Miami model (41.01 kg/km^2^/year) is more than twice that obtained from the NCEAS model (18.61 kg/km^2^/year).

Since prey biomass is derived from the body mass and population density of the species, the expected biomass (*PB*_*E*_) decreases in both the Miami and the NCEAS models, with variations between species. In general, *Rupicapra pyrenaica* shows the greatest reduction in both models (93.53% in Miami and 93.80% in NCEAS), followed by *Castor fiber* (68.98% in Miami and 70.62% in NCEAS). In contrast, *Cervus elaphus* shows the smallest reduction, with values of 35.35 and 38.74% in Miami and NCEAS models, respectively. This is related to species favorability, as species with lower habitat favorability experience a higher reduction in biomass, while those with higher favorability exhibit a lower reduction.

It was observed that body mass influences species’ contribution to prey biomass. Species with high body mass, such as *Stephanorhinus hemitoechus*, despite their low population density (0.32 individuals/km^2^ from the Miami model and 0.11 individuals/km^2^ from the NCEAS model), contribute a greater biomass (Table 6). The opposite occurs for species with lower body mass, such as *Castor fiber*, which, despite having higher population density (6.46 individuals/km^2^ from the Miami model and 2.08 individuals/km^2^ from the NCEAS model), has a low body mass, resulting in a lower biomass contribution to the valley (Table 6).

## Discussion

The upper valley of the Lozoya River during the Late Pleistocene represented a heterogeneous landscape, where elevation and varied vegetation cover created a mosaic of habitats supporting species with different ecological requirements. The nine species from Cueva del Camino analyzed here show a wide predicted distribution across the upper valley of the Lozoya River. Some species, such *Cervus elaphus*, exhibit more uniform distributions, while others, such as * Equus ferus* and *Bos primigenius*, show more variation across different areas. In general, the predicted distribution of these species is constrained by elevation, with altitude being the most selected variable in the models. Higher favorability was found in the intermediate zones (1000–1500 m), which correspond to areas with a more moderate climate, characterized by temperatures between 7 and 9 °C and annual precipitation ranging from 1000 to 800 mm. Additionally, an association was observed between species distribution and their ecology. Woodland species, such as *Capreolus capreolus*, are more prevalent in the intermediate zones of the valley (Fig. 4), which correspond to areas with higher forest density, where forests predominate according to the vegetation reconstructions by Karampaglidis (2015). Simultaneously, favorable habitats were also observed in the lower zones of the valley, where shrubland-like habitats with sub-sclerophyll and hydrophilic vegetation occur (Karampaglidis 2015). This could be explained by the roe deer’s preference for dense forest and scrub areas during the day, and its tendency to seek open areas at night (Kurtén 1968). Similarly, mixed-feeding species such as *Cervus elaphus* and *Dama dama* show greater favorability across the valley. This is consistent with the abundance of these species in the Cueva Camino record, where they represent 70% of the minimum number of individuals (MNI) (Álvarez-Lao et al. 2013). This broad favorability is consistent with the ecological flexibility documented for red deer during the Late Pleistocene. *Cervus elaphus* could exploit both forested and more open habitats depending on local environmental conditions (Sykut et al. 2023). In this context, the high favorability predicted here for *Capreolus capreolus, Cervus elaphus* and *Dama dama* likely reflects their ability to exploit the heterogeneous habitat mosaic of the upper valley of the Lozoya River, rather than a strict association with a single habitat type.

A similar pattern is observed for the beaver, whose distribution aligns with its ecological dependence on aquatic environments, showing preference for the lower parts of the valley. This species is strongly associated with forested riparian habitats and deep, calm, or slow-flowing waters. It can also modify riverine environments locally to create favorable conditions (Holden 2009). Accordingly, the valley-wide low favorability likely reflects the fact that suitable beaver habitat is restricted to particular riparian settings rather than being widespread across the landscape. Incorporating predictors that explicitly capture water availability and vegetation cover (e.g., riparian woodlands and water bodies) would refine SDM predictions of the potential distribution of beavers in the valley.

However, two species, *Stephanorhinus hemitoechus* and *Rupicapra pyrenaica* yielded unexpected results. The rhinoceros shows no clear habitat preference, exhibiting higher densities in elevated areas, contrary to expectations based on its preference for open habitats and steppe grass feeding (Feranec et al. 2010). This could be due to the limited number of fossil records, leading to fewer presence points and potentially affecting model accuracy, as van Proosdij et al. (2016) suggested that 13 occurrences represent the recommended minimum sample size for widespread species. One factor that could influence the rhinoceros’ model is the possibility that some remains at Cueva del Camino were transported from outside the valley. Rhinoceros remains are scarce at the site, accounting for ~ 1.4% of ungulates (Number of Identified Specimens, NISP = 26; MNI = 2), and they consist almost entirely of teeth (Arsuaga et al. 2010, 2012). Because Cueva del Camino has been interpreted primarily as a spotted hyena den (Díez 1993; Arsuaga et al. 2010), a long-distance import scenario would most plausibly require hyenas to transport rhinoceros’ elements from other valleys. However, actualistic studies indicate that transport to dens by *Crocuta crocuta* is on the order of hundreds of meters (mean ~ 0.56 km) (Skinner et al. 1986). Therefore, long-distance imports from other valleys seem unlikely, and the record is more consistent with at least occasional local presence of rhinoceros in the valley. However, selective transport by hominins could still generate isolated non-local occurrences. The chamois shows low habitat favorability in the area, contrary to what would be expected based on its ecology, as higher favorability would be expected in elevated zones, given that it is a species known for its preference for occupying high-altitude habitats (Herrero et al. 1996; Feranec et al. 2010). Similarly to what occurs with the beaver, as a species closely associated with specific environmental conditions, the inclusion of variables that could reflect these habitats, such as slope, could improve SDM predictions. However the expected density maps reveal higher population densities at higher elevations, particularly in the map derived from the NCEAS model (see Fig. 5), possibly reflecting higher productivity (i.e., NPP) in these areas that supports increased presence despite low favorability. Interpreting these SDM-based reconstructions within the climatic framework of MIS 5 in central Iberia provides important context. The 100–80 ka interval (MIS 5c–MIS 5a) encompasses interglacial-to-early glacial variability, during which relatively warm phases alternated with cooler and/or more seasonal conditions. In mountainous landscapes, such as the upper valley of the Lozoya River, this variability is likely to promote strong altitudinal and topographic contrasts. This generated a patchwork of habitats in which herbivores adapted to open spaces and taxa with tolerance of greater cover could coexist on a landscape scale. Thus, the spatial patterns of favorability inferred here reflect how climatic conditions and steep elevational gradients structured the distribution of suitable habitats and the potential prey base available to Neanderthal groups.

This habitat favorability makes expected population densities lower than those that could potentially be achieved. However, these values are not far from what might be found in nature, as values similar to those obtained by Hatton et al. (2015) for similar species are found. These authors studied population densities of different mammal species in African reserves and national parks. Among the species studied by these authors, five could be compared with the species found in the upper valley of the Lozoya River since they belong to the same family. These species are the white rhinoceros (*Ceratotherium simum*) and the black rhinoceros (*Diceros bicornis*), the buffalo (*Syncerus caffer*), the zebra (*Equus burchellii*), and the warthog (*Phacochoerus africanus*). The zebra is the species that shows the most similar values for both Miami and NCEAS models, to those obtained for * Equus ferus* (see Table 5), in Serengeti ecosystem (0.70 individuals/km^2^), and Savuti area of Chobe National Park (0.25 individuals/km^2^). For the buffalo, similar values were found in Hwange National Park (0.69 individuals/km^2^) and Mkomazi Game Reserve (0.23 individuals/km^2^). In the case of *Stephanorhinus hemitoechus*, we found values closer to those obtained from both Miami and NCEAS models in the black rhinoceros, observing densities of 0.37 individuals/km^2^ in Hluhluwe iMfolozi National Park, 0.12 individuals/km^2^ in Kruger National Park and 0.11 individuals/km^2^ in Ngorongoro Crater. For the warthog, most of the values observed by Hatton et al. (2015) are closer to those obtained from the NCEAS model (e.g., 1.37 individuals/km^2^ in Serengeti ecosystem), with the highest observed values for these species (4.73 individuals/km^2^ in Hluhluwe iMfolozi National Park) resembling those obtained with the Miami model. This could be related to the fact that for these species, the Miami model is overestimating the population density (see Supplementary Material).

In the case of the cervid species, we observed that the densities in our study area are generally lower than those recorded by Ripple and Beschta (2012), although not for both Miami and NCEAS models. These authors studied the population densities of ungulate species across different locations in Northern Hemisphere Forest ecosystems. Taking *Cervus elaphus* as a reference, we compared *Dama dama* and *Capreolus capreolus* with the two next smallest body mass species, which are *Rangifer tarandus* and *Odocoileus virginianus*, respectively. This comparison is intended only as a body-mass benchmark, and we acknowledge the ecological differences among these taxa. We found similar values to those obtained for both models (Miami and NCEAS) only for *Capreolus capreolus* in Voyageurs National Park (8.4 individuals/km^2^) and Papineau-Labelle (3 individuals/km^2^) (see Table 5). In the case of *Rangifer tarandus*, we found a single value in Laplandsky of 1.4 individuals/km^2^, similar to the value obtained with the NCEAS model for *Dama dama* (see Table 5). Finally, the lowest value observed for *Cervus elaphus* is 3.3 individuals/km^2^ in Wind Cave National Park, closer to the value obtained with the Miami model. In general, we observed that the densities in our study area are lower than those recorded by Ripple and Beschta (2012). Because Ripple and Beschta (2012) restricted their dataset to non-migratory populations, this comparison is not strictly equivalent if cervids in the upper valley of the Lozoya River were seasonally mobile (e.g., elevational migration). Seasonal elevational movements could reduce time-averaged local densities within the study area relative to resident populations. *Cervus elaphus*, for example, is known to migrate altitudinally in mountainous regions, moving from low-elevation winter ranges to high-elevation summer ranges, which can generate seasonal changes in local densities (Kropil et al. 2015; Smolko et al. 2018). However, because our SDMs are based on time-averaged environmental predictors and fossil occurrences pooled within 80–100 ka, we cannot model seasonality explicitly. Therefore, we interpret our estimates as an annual-average baseline of potential distribution and prey biomass across the valley.

The difference between potential and expected population density is reflected in the prey biomass, which shows that considering habitat favorability in the study area plays a key role in estimating the biomass of each species, with the expected biomass being lower than what could potentially be achieved, especially for those species that have less habitat favorability, such as *Rupicapra pyrenaica*. This is evident when comparing the variation between potential biomass (*PB*_*P*_) and expected biomass (*PB*_*E*_) for *Dama dama* and *Rupicapra pyrenaica*, observing that the first species has a higher habitat favorability (mean favorability = 0.589), and therefore, the percentage of biomass reduction is lower (42.22 and 44.15%, in Miami and NCEAS, respectively) compared to *Rupicapra rupicapra*, which is the species with the lowest habitat favorability (mean favorability = 0.067), leading to a greater reduction in biomass (93.53 and 93.80%, in Miami and NCEAS, respectively).

This potential biomass (*PB*_*P*_) can be compared to the carrying capacity studied by Molino et al. (2025) for the same ecosystem, with a value of 2037.40 kg/km^2^/year, since that study applied maximum density for all species. The estimates by Molino et al. (2025) are based on current population density data, whereas in the present study, population densities were estimated from NPP values, which in some cases result in higher productivity than what is currently observed. When comparing the values, we observe that in the case of the potential biomass obtained through the Miami model, the values are much higher (4140.17 kg/km^2^/year) than those found by these authors (see Table 6). In contrast, when compared with the potential biomass (*PB*_*P*_) value obtained from the NCEAS model, the values are lower (1409.83 kg/km^2^/year). This could reflect the overestimation of the Miami model as well as the underestimation of the NCEAS model (see Supplementary Material).

When we compared the expected prey biomass (*PB*_*E*_) values with those reported by Hatton et al. (2015) for African ecosystems, we found prey biomass values closer to those obtained with the Miami model than with the NCEAS model (see Table 6). The Serengeti (2000.5 kg/km^2^) shows values similar to those of the Miami model, while the value closest to that of NCEAS is the one observed in Hwange National Park (608.3 kg/km^2^). It should be noted, however, that these authors do not consider large herbivores like the rhinoceros, so higher prey biomass values could be expected for those locations.

In the case of predator biomass, similar comparisons were made. When comparing the potential predator biomass (*DB*_*P*_) values with those obtained by Molino et al. (2025) (24.48 kg/km^2^/year), we observe that the value estimated using the NCEAS model (18.61 kg/km^2^) is closer to the one reported by these authors, while the Miami model produces a much higher value (41.01 kg/km^2^) (Table 6). Likewise, when comparing expected predator biomass (*DB*_*E*_) to values from African national parks previously mentioned, we observe lower predator biomass in those parks than in our study. Parks where prey biomass values are comparable to those predicted by the Miami model, such as the Serengeti National Park, report predator biomass values of 33.8 kg/km^2^. In Hwange National Park, where the prey biomass aligns more closely with the NCEAS estimate, predator biomass is 7.5 kg/km^2^. However, when we compare the values of the prey-predator biomass ratio, we observe that the results from the upper valley of the Lozoya River (81.15 kg prey/kg predator and 60.09 kg prey/kg predator, for Miami and NCEAS, respectively) show similarities with several African ecosystems, such as Hwange National Park (81.37 kg prey/kg predator), Nairobi National Park (81.36 kg prey/kg predator) and Kruger National Park (80.63 kg prey/kg predator), on the one hand, and Kruger National Park (63.88 kg prey/kg predator) and Serengeti (59.27 kg prey/kg predator), on the other hand, suggesting that similar predator–prey dynamics may have existed in the upper valley of the Lozoya River.

From the expected predator biomass, we can estimate the number of Neanderthals that the environment could support. As a bounding scenario, considering Neanderthals as the only secondary consumer in the area and assuming a body mass of 71 kg (Will et al. 2017), we estimated a Neanderthal population of 140 and 62 individuals using the Miami and NCEAS models, respectively, based on the expected predator biomass (22.85 kg/km^2^ and 10.14 kg/km^2^, for the Miami and NCEAS models, respectively) and a total area of 434.58 km^2^. Applying this approach to the CC value of Molino et al. (2025) (2037.40 kg/km^2^/year), i.e., considering Neanderthals as the only secondary consumer in the area (434.58 km^2^), an average of 148 individuals was obtained. The value from the NCEAS model seems to be closer to what would be expected, since the approach of Molino et al. (2025) assumed a homogeneous distribution of prey species in the upper valley of the Lozoya River. These authors estimated the number of Neanderthals that could be supported by considering the other secondary consumer species present in the upper valley of the Lozoya River, yielding a number of 34 Neanderthals. This approach is more realistic than assuming Neanderthals were the only secondary consumers. Molino et al. (2025) estimated the relative biomass of each secondary consumer using the product of body mass and population density. They took body mass values from Rodríguez-Gómez et al. (2017b) for carnivores and from Will et al. (2017) for Neanderthals (71 kg). They estimated density values using the allometric relationship between body mass and density from Damuth (1993) for carnivores. For Neanderthals, they used the density based on ethnographic averages for hunter-gatherers (0.25 individuals per km^2^; Marlowe 2005). Following this framework, we estimated the relative contribution of Neanderthals to total secondary-consumer biomass. Based on the predator biomass values of 22.85 kg/km^2^ and 10.14 kg/km^2^ (Miami and NCEAS), the estimated Neanderthal biomass would be 5.29 and 2.35 kg/km^2^, respectively. Dividing by Neanderthal body mass (71 kg) yields densities of 0.075 and 0.033 ind/km^2^, respectively, corresponding to 33 and 14 (Miami and NCEAS, respectively) individuals when scaled to the area of 434.58 km^2^ (Karampaglidis 2015). These values also suggest that the NCEAS model is more consistent with the range of estimates reported by Molino et al. (2025), who calculated a maximum population size based on a uniform distribution across the valley. Overall, we observed that the values obtained using the Miami model are higher than those from the NCEAS model, which could reflect an overestimation by the Miami model as well as an underestimation by the NCEAS model (see Supplementary Material). Therefore, we could argue that the NCEAS model provides values closer to what would be expected, since the work by Molino et al. (2025) refers to maximum population densities, and the values from the Miami model exceed or equal these in all cases. These values suggest that the upper valley of the Lozoya River could have supported repeated, though not necessarily continuous, Neanderthal presence. It should be noted that, while this study refines the approach of Molino et al. (2025), the resulting values should be understood as potential demographic estimates, since they are derived from carrying-capacity calculations that represent potential ecological conditions for the prey community. In this context, the relatively low estimated numbers suggest that these groups may have used the area as a hunting ground while also exploiting surrounding areas. This interpretation is also consistent with growing evidence that Neanderthal communities were often small, socially variable, and spatially dispersed. El Sidrón and Le Rozel suggest groups of limited size and differing composition, while genomic data from the Altai indicate small communities with close kin ties, female-biased migration, and, in some cases, marked isolation (Lalueza-Fox et al. 2011; Ríos et al. 2019; Duveau et al. 2019; Skov et al. 2022; Massilani et al. 2026). Thus, the low population estimates obtained here are best understood as compatible with repeated use of the upper valley of the Lozoya River by small groups, rather than by large or continuously resident populations. This interpretation is consistent with discussions of Neanderthal settlement in the interior of the Iberian Peninsula, which emphasize low-density, highly mobile groups and short-term occupations (Sánchez-Romero et al. 2017; Sala et al. 2020; Moclán et al. 2021). The valley’s enclosed topography, steep slopes, and altitudinal gradients would have created a mosaic of habitats, influencing prey distribution and providing strategic vantage points for resource exploitation, transforming this rugged landscape into a dynamic ecological niche. In addition, the possibility that the prey were migratory individuals with high mobility in the exit zone of the valley could have influenced hunting strategies, with Neanderthals targeting areas of high prey movement at certain times of the year. This interpretation is supported by isotopic evidence indicating seasonal mobility in red deer during the Pleistocene. For example, Barakat et al. (2023) showed that red deer occupied distinct seasonal ranges, being more accessible near archaeological sites during autumn and winter, whereas summer hunting could require movements of up to ~ 20 km. Such patterns suggest that locally high prey availability may reflect seasonal concentration rather than year-round presence. The seasonal mobility of key prey species, such as red deer (*Cervus elaphus*), which can undergo altitudinal migration between winter and summer ranges (Kropil et al. 2015; Smolko et al. 2018), may have generated predictable seasonal pulses in movement and locally higher densities along preferred routes. In a topographically confined system such as the upper valley of the Lozoya River, these movements may have been funneled toward the valley outlet. This could have favored short-duration Neanderthal visits timed to periods of peak prey movement.

Our study proposes a framework based on prey biomass to estimate Neanderthal population potential in the past. This framework complements other approaches that infer population parameters from land use analogies and more general behavioral assumptions (e.g., Churchill et al. 2016). For future approaches, it would be interesting to consider predator–prey relationships and competition among secondary consumers, as competition among species could influence how they could potentially distribute in the environment (Thuiller et al. 2004). One way to do this will be to use the PSEco model (Rodríguez-Gómez et al. 2024) to estimate how many Neanderthals the valley could support based on the estimated prey biomass, taking into account competition with carnivores.

In summary, from a methodological perspective, the present study shows a good approximation for understanding the paleoecosystem of the Lozoya Valley. Its validity is supported by the observation that, in modern ecosystems, prey biomass typically does not reach its maximum potential (Fig. S2, Supplementary Material). This provides a new perspective on estimating the carrying capacity of past ecosystems, where it is shown that ecosystems tend not to reach maximum carrying capacity, and therefore, incorporating the potential distribution of species is of great importance to adjust these values to those closer to what would be expected. However, we identified some limitations that could affect SDMs and their results. Among these is the need to use environmental variables to model the potential distributions of species, which is challenging in the case of past periods, as variables such as climate, topography, or vegetation cover can be challenging to obtain and adjust to the desired resolution. A similar issue arises with species occurrence data, as the lack of enough fossil records for one or more species, such as *Stephanorhinus hemitoechus*, can affect the reliability of the resulting SDM.

In regard to Neanderthal settlements, our findings suggest that the upper valley of the Lozoya River was a challenging landscape for Neanderthal populations. The valley’s rugged topography, steep altitudinal gradients, and variable seasonal climate created an uneven and fragmented ecological landscape. While certain areas offered favorable conditions for prey species, the overall ecological capacity was limited, supporting relatively few Neanderthal individuals. These conditions likely led them to develop strategies to exploit localized resources and integrate the valley into broader seasonal mobility patterns. Our study contributes to the growing body of evidence indicating that mountain environments in the central Iberian Peninsula posed significant ecological constraints that affected the demographic limits and behavioral flexibility of hunter-gatherer groups. By quantifying these constraints using ecological models, we provide a framework for understanding how large mammal communities influenced Neanderthal persistence in Late Pleistocene ecosystems.

## Conclusions

The results of this study demonstrate the effectiveness of species distribution models (SDMs) in reconstructing the spatial distribution of large herbivores in late Pleistocene ecosystems. The models successfully captured the environmental preferences of different species, closely matching their known ecological requirements. In addition to mapping habitat favorability, SDMs proved valuable for estimating herbivore biomass because they consider the gap between potential and realized biomass observed in modern ecosystems. Compared to previous work in the valley, which relied on more simplified and spatially uniform assumptions, this approach provides a more realistic basis for assessing the carrying capacity of the upper valley of the Lozoya River during the Late Pleistocene. Considering the estimated prey biomass, the expected number of Neanderthals in the region, and the geographic characteristics of the area, the valley was part of their recurring hunting and resource exploitation area. Future research should incorporate species interactions and trophic relationships into distribution models. Incorporating these dynamic ecological processes would yield more accurate, ecologically realistic reconstructions of past ecosystems and improve our understanding of the environmental contexts that shaped Neanderthal occupation in central Iberian Peninsula.

## Supplementary Information

Below is the link to the electronic supplementary material.

Supplementary file1 (DOCX 180 KB)

## Data Availability

Data are provided in the manuscript and supplementary materials.
