# Supplementary material for: Linking prey biomass and Neanderthal population in a challenging upland landscape in the Late Pleistocene: the upper valley of the Lozoya River (Spain)
Source: Landsc Ecol. 2026 Apr 21;41(7):116. doi: 10.1007/s10980-026-02350-x (PMC13315265; doi:10.1007/s10980-026-02350-x)
Supplement: Supplementary file 1 — Supplementary file1 (DOCX 180 KB) [file 10980_2026_2350_MOESM1_ESM.docx]

**Supplementary Material**

1. Archaeologsical and paleontological sites containing remains of the selected species found at the Cueva del Camino site

| **Site** | **Layer** | **Longitude** | **Latitude** | **Reference** |
| --- | --- | --- | --- | --- |
| Abri Bourgeois-Delaunay | 7 | 0,447 | 45,667 | Jaubert 2002; Couchoud 2006; Vermeersch 2025 |
| Abri du Maras | 5 | 44,313 | 4,559 | Moncel and Michel 2000; Marín et al. 2020 |
| Arbreda | I | 2,75 | 42,16 | Yokoyama et al. 1987; Kehl et al. 2014 |
| Arbreda | J | 2,75 | 42,16 | Yokoyama et al. 1987; Kehl et al. 2014 |
| Arbreda | K | 2,75 | 42,16 | Yokoyama et al. 1987; Kehl et al. 2014 |
| Arriaga II | Mz-5 | -3,862 | 40,312 | Sesé and López-Martínez 2013; Silva et al. 2013 |
| Artazu VII |  | -2,529 | 43,074 | Suárez-Bilbao et al. 2016; Puzachenko et al. 2021 |
| Asprochaliko | 18 | 20,84 | 39,24 | Roditi and Starkovich 2022 |
| Baume-Vallée, Abri Laborde | Units 0, 1 and 2 | 3.883 | 44.967 | Raynal and Decroix 1986; Fiore et al. 2005; Raynal et al. 2005; Daujeard et al. 2012 |
| Bérigoules | I | 5,2547 | 43,98425 | Richter et al. 2007; Vermeersch 2025 |
| Bérigoules | II | 5,2547 | 43,98425 | Richter et al. 2007; Vermeersch 2025 |
| Bisnik Cave | 10 | 19,66 | 50,43 | Cyrek et al. 2010 |
| Bollschweil | 1 | 7,78 | 47,93 | Rink et al. 2002 |
| Buena Pinta | 3 | -3,82037 | 40,9265 | Torres et al. 2014 |
| Can Costella |  | 1,66542 | 41,45883 | Bonet Avalos et al. 1993; Estévez et al. 1996 |
| Can Garriga |  | 2,83157 | 42,0294 | Garcia 2015 |
| Carihuela | VIIb-1 | -3,43312 | 37,444077 | Carrión et al. 2019 |
| Carihuela | VIIb-2 | -3,43312 | 37,444077 | Carrión et al. 2019 |
| Carihuela | CIII 3-X | -3,43312 | 37,444077 | Carrión et al. 2019 |
| Caverna delle Fate |  | 8,3675 | 44,19583 | Valensi and Psathi 2004; Vermeersch 2025 |
| Cedres | II | 5,71667 | 43,33333 | Valladas et al. 1999 |
| Cedres | III | 5,71667 | 43,33333 | Valladas et al. 1999 |
| Chez-Pinaud I | 22 | -0,41926 | 45,43733 | Niven et al. 2012; Richter et al. 2013 |
| Corazón | 2 | -4,2682 | 42,74503 | Sanchez Yustos et al. 2011 |
| Cotte Saint Brelade | III | -2,02 | 49,185 | Bates et al. 2013; Kandel et al. 2023 |
| Cotte Saint Brelade | IV | -2,02 | 49,185 | Bates et al. 2013; Kandel et al. 2023 |
| Cova Eirós | 3 | -7,21015 | 42,75224 | Rodríguez-Álvarez et al. 2011; Vermeersch 2025 |
| Cova Negra | 12 | -0,53 | 38,98 | Arsuaga et al. 2007, 2012; Puzachenko et al. 2021 |
| Cueva del Camino | 5 | -3,862 | 40,909 | Arsuaga et al. 2011, 2012; Baquedano et al. 2014 |
| Denisova Cave Main Chamber | 14 | 84,67611 | 51,3975 | Jacoks et al. 2019; Kandel et al. 2023; Vermeersch 2025 |
| El Castillo | 22 | -3,97 | 43,29 | Martín-Perea et al. 2022; Vermeersch 2025 |
| Estanque de Tormetas de Butarque | H-02 | -3,76062 | 40,34098 | Laplana et al. 2015; López-Recio et al. 2018 |
| Estret de Trago | UA2 | 0,72 | 41,87 | Casanova i Martí et al. 2009; Vermeersch 2025 |
| Figueira Brava | FB4-IH8 | -8,96611 | 38,47065 | Zilhão et al. 2020 |
| Furninha |  | -9,4008 | 39,356 | Bicho and Cardoso 2010; Vermeersch 2025 |
| Galería de las Estatuas | LU1–LU4 | -3,51732 | 42,35038 | Arsuaga et al. 2017; Demuro et al. 2019; Moreno et al. 2022 |
| Ganovce | E | 20,33 | 49,03 | Sabol et al. 2017; Vermeersch 2025 |
| Geula Cave | B3 | 35 | 32,8 | Barzilai et al. 2022 |
| Grotta dei Moscerini |  | 13,485 | 41,236 | Stiner 1993; Villa et al. 2020 |
| Grotta Maggiore di San Bernardino | IV | 11,55 | 45,42 | López-García et al. 2019; Vermeersch 2025 |
| Grotte des Barasses II | 7 | 4,37 | 44,5167 | Rufà et al. 2018; Richard et al. 2021 |
| Gruta Nova da Columbeira | 8 | -9,2 | 39,3 | Zilhão et al. 2011 |
| Hallera Avenue | B | 16,99 | 51,09 | Wiśniewski et al. 2024; Vermeersch 2025 |
| Ignatevskaya cave |  | 57,76667 | 54,88333 | Kosintsev and Bachura 2013; Dublyansky et al. 2021 |
| Kabazi II | III/2, stratum 11 | 34,03 | 44,83 | Patou-Mathis and Chabaï 2003; Vermeersch 2025 |
| Kabazi II | III/3 stratum 11 | 34,03 | 44,83 | Patou-Mathis and Chabaï 2003; Vermeersch 2025 |
| Karain Cave | AH20-I.7-H | 30,57 | 37,08 | Rink et al. 1994 |
| Karain Cave | AH25-III.2-F | 30,57 | 37,08 | Rink et al. 1994 |
| Kulna Cave | 12a | 16,74 | 49,41 | Nejman et al. 2011 |
| Kulna Cave | 14 | 16,74 | 49,41 | Nejman et al. 2011 |
| La Ferrassie_Grand Abri | A | 0,94 | 44,95 | Guérin et al. 2015; Vermeersch 2025 |
| Le Rozel |  | -1,84263 | 49,47129 | Stoetzel et al. 2016; Mercier et al. 2019 |
| Madonna dell Arma | III-IV | 7,85 | 43,83 | Kaniewski et al. 2005; Cauche 2007; Vermeersch 2025 |
| Moula Guercy | XV | 4,85 | 44,88 | Willmes et al. 2016; Grün and Stringer 2023; Vermeersch 2025 |
| Mutzig-rain | C3 | 7,44442 | 48,53958 | Koehler et al. 2021; Bahain et al. 2024 |
| Mutzig-rain | C5 | 7,44442 | 48,53958 | Koehler et al. 2021; Bahain et al. 2024 |
| Mutzig-rain | C5/7A | 7,44442 | 48,53958 | Koehler et al. 2021; Bahain et al. 2024 |
| Mutzig-rain | C7A | 7,44442 | 48,53958 | Koehler et al. 2021; Bahain et al. 2024 |
| Mutzig-rain | C7C | 7,44442 | 48,53958 | Koehler et al. 2021; Bahain et al. 2024 |
| Mutzig-rain | C8/9 | 7,44442 | 48,53958 | Koehler et al. 2021; Bahain et al. 2024 |
| Mutzig-rain | C9 | 7,44442 | 48,53958 | Koehler et al. 2021; Bahain et al. 2024 |
| Mutzig-rain | C10 | 7,44442 | 48,53958 | Koehler et al. 2021; Bahain et al. 2024 |
| Neumark-Nord | 2/0 | 11,9 | 51,32 | Laurat and Brühl 2021 |
| Oliveira | 8 | -8,61361 | 39,50639 | Zilhão et al. 2021; Kandel et al. 2023; Vermeersch 2025 |
| Oliveira | 11 | -8,61361 | 39,50639 | Zilhão et al. 2021; Kandel et al. 2023; Vermeersch 2025 |
| Payre^1^ | D | 4,73611 | 44,73167 | Foury et al. 2016; Kandel et al. 2023; Vermeersch 2025 |
| Pech de l Azé II | 4B | 1,25 | 44,86 | Jacobs et al. 2016; Vermeersch 2025 |
| Pecheurs | Sector 4 | 4,21 | 44,41 | Moncel and Lhomme 2007; Vermeersch 2025 |
| Pešturina cave | 4a | 21,9 | 43,167 | Blackwell et al. 2014; Puzachenko et al. 2021; Mihailović et al. 2022; Vermeersch 2025 |
| Pešturina cave | 4c | 21,9 | 43,16667 | Blackwell et al. 2014; Puzachenko et al. 2021; Mihailović et al. 2022 Vermeersch 2025 |
| Preresa |  | -3,589 | 40,303 | López-Recio et al. 2018; Vermeersch 2025 |
| Ramandils | V | 3,0346 | 43,00618 | Rush et al. 2019; Vermeersch 2025 |
| Rinoceront | I | 1,960833333 | 41,27361111 | Daura et al. 2015 |
| Riparo Mochi | I | 7,53478 | 43,78406 | Frouin et al. 2022; Kandel et al. 2023; Vermeersch 2025 |
| Roc de Marsal | 4 | 0,96705 | 44,90636 | Goldberg et al. 2012; Guérin et al. 2017 |
| Roc de Marsal | 8 | 0,96705 | 44,90636 | Goldberg et al. 2012; Guérin et al. 2017 |
| Roc de Marsal | 9 | 0,96705 | 44,90636 | Goldberg et al. 2012; Guérin et al. 2017 |
| Scladina | 3 | 5,02 | 50,48 | Puzachenko et al. 2021; Vanhof et al. 2024 |
| Tabun Cave | B | 34,97 | 32,67 | Grün and Stringer 2000; Kandel et al. 2023 |
| Tata |  | 18,32853 | 47,65526 | Borel et al. 2017; Vermeersch 2025 |
| Vanguard Cave |  | -5,34167 | 36,121389 | Carrión et al. 2018; Doerschner et al. 2019; Vermeersch 2025 |
| Vindija | H/I | 16,08 | 46,3 | Karavanić et al. 2021; Vermeersch 2025 |
| Vindija | H | 16,08 | 46,3 | Karavanić et al. 2021; Vermeersch 2025 |
| Zwoleń | IV | 21,588 | 51,355 | Wojtal and Patou-Mathis 2003; Vermeersch 2025 |

^1^Payre Layer D was retained on the basis of the biostratigraphic attribution of Foury et al. (2016), who place this level in MIS 5c or MIS 5a, despite the complexity of the available radiometric chronology.

1. Evaluation of NPP Estimation Models

To estimate the population densities of each species, we used two models to calculate the Net Primary Productivity (NPP) (i.e., the Miami model (Lieth 1975) and the NCEAS model (Del Grosso et al. 2008)). Both models rely on mean annual temperature and annual precipitation values to estimate NPP. In the case of the NCEAS model, two different estimates are proposed depending on whether the ecosystem is non-tree dominant or tree dominant. In our case, we selected the estimate for tree-dominant ecosystems, as it provides higher NPP values, and we preferred to use higher values that could act as an upper limit in the estimations.

The objective is to assess which of the two models best fits the expected NPP values in the Upper Lozoya Valley by comparing the current values for this environment, obtained using these models, with the reference value proposed by the EEA (European Environment Agency 2023). To obtain the NPP values from the Miami and NCEAS models, we used mean annual temperature and annual precipitation values from WorldClim (Fick and Hijmans 2017). Subsequently, we obtained the values from EEA for the year 2000, which were used as a reference for comparison.

We observed that the Miami model estimates NPP values higher than those proposed by the EEA, whereas the NCEAS model estimates lower values (Table S1). This could indicate an overestimation of NPP by the Miami model and an underestimation by the NCEAS model, as shown in Figure S1, where data points above the 1:1 line indicate overestimation, while those below it indicate underestimation. This pattern aligns with findings from Del Grosso et al. (2008), who reported that the Miami model tends to overestimate NPP. Similarly, the NCEAS model may be underestimating values, as its NPP estimates are based on specific biomes.

Therefore, we could expect the NPP value for the Upper Lozoya Valley to fall between the values obtained from the Miami and NCEAS models.

**Table S1**. NPP values obtained from the Miami and NCEAS models and those proposed by the EEA, expressed in g m^-2^ year^-1^, for the Upper Valley of the Lozoya river.

|  | **NPP (g/m^2^/year)** |
| --- | --- |
| Modelo Miami | 1012.12 |
| Modelo NCEAS | 441.02 |
| EEA | 660.55 |


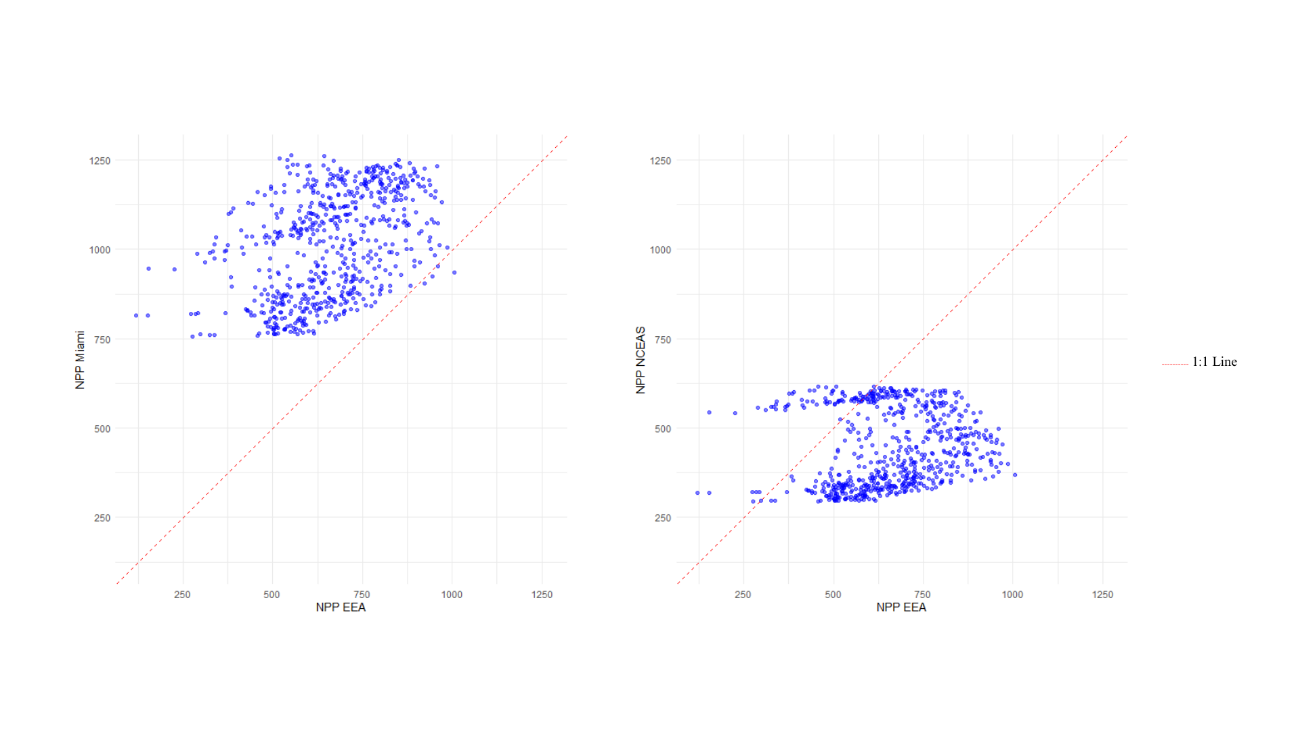


**Figure S1**. Distribution of EEA NPP values versus those obtained with the Miami and NCEAS models in the Upper Lozoya Valley.

1. Methodology Validation

With the validation of the methodology, we aim to assess whether the observed reduction in prey biomass in the Lozoya Valley resembles patterns observed in current ecosystems. The ecosystems selected for comparison were those described by Hatton et al. (2015) for Africa, India, and North America, as well as those observed by Rodríguez et al. (2014) for different regions worldwide. Biomass reduction is defined as the proportion of potential biomass not realized in the observed biomass:

Biomass reduction = (B – b) / B , (1)

where B represents the potential biomass derived from equation (2), and b is the observed biomass.

To estimate the potential biomass of current ecosystems, we first calculated the NPP using two empirical models, the Miami model (Lieth 1975) and the NCEAS model (Del Grosso et al. 2008). Both models use mean annual temperature and annual precipitation as predictors, which were obtained from the WorldClim (Fick and Hijmans 2017) variables bio01 and bio12. The NCEAS model accounts for differences among biome types, distinguishing between tree-dominated and non-tree-dominated ecosystems. We used the NPP estimates for tree-dominated ecosystems, as these values were higher than those for non-tree-dominated ecosystems, considering them as the maximum potential NPP. Subsequently, the potential biomass (B) was calculated for each NPP model as the total herbivore biomass (THB) that the ecosystem could support, using the predictive equation proposed by Vidal-Cordasco et al. (2022):

THB = 10^ (1.401 × log10[NPP] − 0.642) , (2)

where the values of THB and NPP are expressed in g m^-2^ year^-1^.

In the case of the Lozoya Valley, the observed biomass values used were those of expected biomass (*PB_E_*) obtained in the present study. To increase the dataset, potential biomass (*PB_P_*) and observed biomass (*PB_E_*) values were taken for 50 random points within the Upper Lozoya Valley area.

It was observed that at some locations, no reduction in biomass occurred, and instead, the observed biomass exceeded the potential biomass. This could be because, being reserves and national parks, nutritional support may be occurring in some of them, leading to observed biomass surpassing the potential biomass. Therefore, these locations were excluded from the analysis, leaving only those where a reduction in biomass was observed, leading to the number of locations presented in Table S2 as the “filtered number”.

The results show that with the NCEAS model, we obtained biomass reductions similar to those observed in current herbivore communities (Fig. S2B). On the other hand, with the Miami model, current ecosystems exhibited greater reductions in biomass than those observed in the Lozoya Valley (Fig. S2A). This could reflect an overestimation by the Miami model, which leads to much higher potential biomass values, thus increasing the difference with observed biomass. Nevertheless, we observed that with the NCEAS model, there were fewer locations where the potential biomass exceeded the observed biomass, compared to the Miami model, where the number of such locations was higher, possibly indicating an underestimation of biomass with the NCEAS model (Table S2). However, when comparing only North America, as it is the only region where all locations are included in both NPP models, we observed that the NCEAS model provides biomass reductions more similar to those observed in these locations compared to the Miami model (Fig. S2).

Therefore, the methodology followed in this study estimates herbivore biomass reduction that closely aligns with what is observed in current ecosystems, highlighting the importance of considering the potential distribution of species in the study area when estimating prey biomass.

**Table S2**. Number of locations used for the analysis. The original number refers to the number of locations in each dataset, while the filtered number represents the remaining observations after keeping only the locations where there is a biomass reduction.

|  | Original number | Filtered number | |
| --- | --- | --- | --- |
|  |  | Miami model | NCEAS model |
| Africa | 46 | 37 | 11 |
| India | 88 | 88 | 61 |
| North America | 52 | 52 | 52 |
| Global | 94 | 59 | 35 |


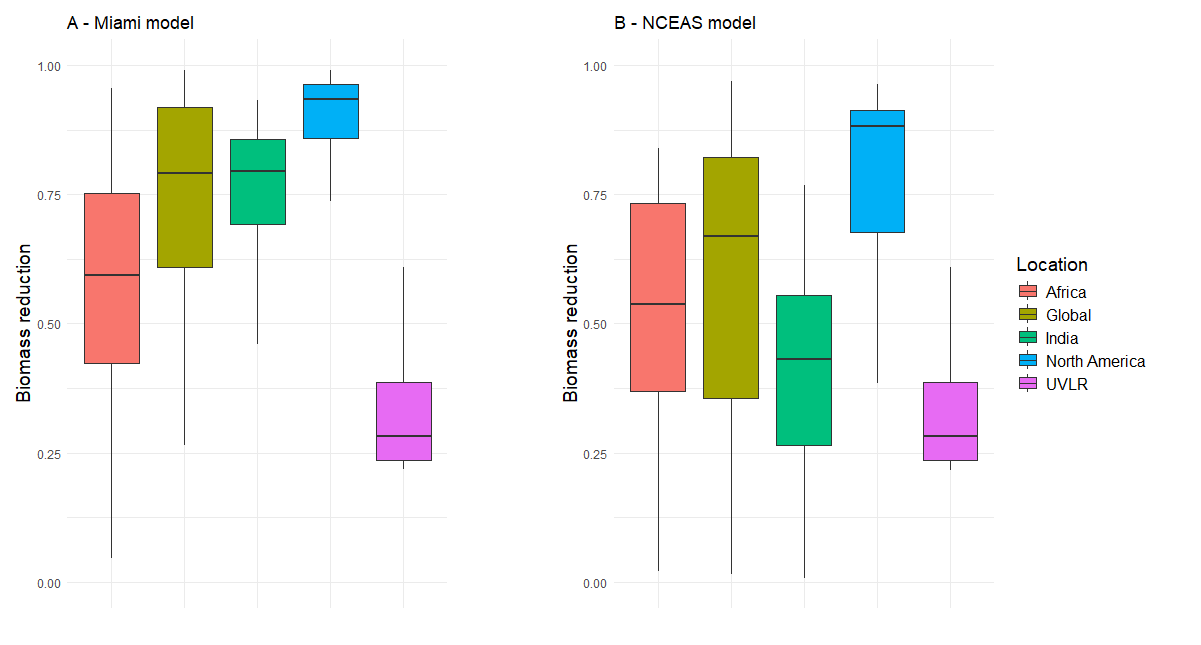


**Figure S2**. Comparison of biomass reduction across models and locations

**References**

Arsuaga JL, Baquedano E, Pérez-González A (2011) Neandernthal and carnivore occupations in Pinilla del Valle sites (Community of Madrid, Spain). Archaeopress 111–119

Arsuaga JL, Baquedano E, Pérez-González A, Sala N, Quam RM, Rodríguez L, García R, García N, Álvarez-Lao DJ, Laplana C, Huguet R, Sevilla P, Maldonado E, Blain HA, Ruiz-Zapata MB, Sala P, Gil-García MJ, Uzquiano P, Pantoja A, Márquez B (2012) Understanding the ancient habitats of the last-interglacial (late MIS 5) Neanderthals of central Iberia: Paleoenvironmental and taphonomic evidence from the Cueva del Camino (Spain) site. Quat Int 275: 55–75. https://doi.org/10.1016/j.quaint.2012.04.019

Arsuaga JL, Gómez-Olivencia A, Sala N, Martínez-Pillado V, Pablos A, Bonmatí A, Pantoja-Pérez A, Lira-Garrido J, Alcázar De Velasco A, Ortega AI, Cuenca-Bescós G, García N, Aranburu A, Ruiz-Zapata B, José Gil-García M, Rodríguez-Álvarez XP, Ollé A, Mosquera M (2017) Evidence of paleoecological changes and Mousterian occupations at the Galería de las Estatuas site, Sierra de Atapuerca, northern Iberian plateau, Spain. Quat res 88:345–367. https://doi.org/10.1017/qua.2017.46

Arsuaga JL, Villaverde V, Quam R, Martínez I, Carretero JM, Lorenzo C, Gracia A (2007) New Neandertal remains from Cova Negra (Valencia, Spain). Journal of Human Evolution 52:31–58. https://doi.org/10.1016/j.jhevol.2006.07.011

Bahain J-J, Tombret O, Garbé L, Falguères C, Koehler H, Wegmüller F (2024) ESR/U-series dating of palaeontological remains from the Neanderthal site of Mutzig-Rain (Alsace, France). Quat Geochronol 81:101517. https://doi.org/10.1016/j.quageo.2024.101517

Baquedano E, Márquez Mora B, Laplana Conesa C, Arsuaga Ferreras JL, Pérez González A (2014) The archaeological sites at Pinilla del Valle: (Madrid, Spain). In: Pleistocene and Holocene hunter-gatherers in Iberia and the Gibraltar strait: the current archaeological record. Fundación Atapuerca, pp 577–584

Barzilai O, Abulafia T, Shemer M, May H, Orbach M, Frumkin A, Yeshurun R, Sarig R, Porat N, Hershkovitz I (2022) Rediscovering Geula Cave: A Middle Paleolithic cave site in northern Mt. Carmel, Israel. Quaternary International 624:181–197. https://doi.org/10.1016/j.quaint.2021.03.007

Bates M, Pope M, Shaw A, Scott B, Schwenninger J-L (2013) Late Neanderthal occupation in North-West Europe: rediscovery, investigation and dating of a last glacial sediment sequence at the site of La Cotte de Saint Brelade, Jersey. Journal of Quaternary Science 28:647–652. <https://doi.org/10.1002/jqs.2669>

Bicho N, Cardoso JL (2010) Paleolithic occupations and lithic assemblages from Furninha Cave, Peniche (Portugal). Zephyrus 66: 17–38.

Blackwell B, Chu S, Chaity I, Huang YEW, Mihailović D, Roksandic M, Dimitrijević V, Blickstein J, Huang A, Skinner AR (2014) ESR dating ungulate tooth enamel from the Mousterian layers at Pešturina, Serbia. In: Mihailović D (ed) Palaeolithic and Mesolithic research in the Central Balkans. Belgrade: Serbian Archaeological Society, pp 21–38

Bonet Avalos A, Piqué Huerta R (1995) Dinámica paleoambiental y paleoclimática de la cuenca del Mediona-Riudevitlles (Alt Penedès, Barcelona): análisis antracológico y malacológico de los yacimientos de Can Costella y Mediona I. In: Aleixandre Campos T, Pérez-González A (eds) Reconstrucción Paleoambiental y Cambios Climáticos durante el Cuaternario, pp. 363–374.

Borel A, Dobosi V, Moncel M-H (2017) Neanderthal’s microlithic tool production and use, the case of Tata (Hungary). Quat Int 435:5–20. https://doi.org/10.1016/j.quaint.2015.09.102

Carrión JS, Fernández S, Jiménez-Arenas JM, Munuera M, Ochando J, Amorós G, Ponce de León M, Zollikofer C, Martín-Lerma I, Toro-Moyano I, Hajdas I, Walker MJ (2019) The sequence at Carihuela Cave and its potential for research into Neanderthal ecology and the Mousterian in southern Spain. Quat Sci Rev 217:194–216. https://doi.org/10.1016/j.quascirev.2019.04.012

Carrión JS, Ochando J, Fernández S, Blasco R, Rosell J, Munuera M, Amorós G, Martín-Lerma I, Finlayson S, Giles F, Jennings R, Finlayson G, Giles-Pacheco F, Rodríguez-Vidal J, Finlayson C (2018) Last Neanderthals in the warmest refugium of Europe: Palynological data from Vanguard Cave. Rev Palaeobot Palynol 259:63–80. <https://doi.org/10.1016/j.revpalbo.2018.09.007>

Casanova i Martí J, Martínez Moreno J, Mora Torcal R, de la Torre I (2009) Stratégies techniques dans le Paléolithique Moyen du sud-est des Pyrénées. L’Anthropologie 113: 313–340. <https://doi.org/10.1016/j.anthro.2009.04.004>

Cauche D (2007) Les cultures moustériennes en Ligurie italienne : analyse du matériel lithique de trois sites en grotte. L’Anthropologie 111: 254–289. https://doi.org/10.1016/j.anthro.2007.05.002

Couchoud I (2006) Étude pétrographique et isotopique de spéléothèmes du Sud-Ouest de la France formés en contexte archéologique: contribution à la connaissance des paléoclimats régionaux du stade isotopique 5. PhD thesis, Université Bordeaux 1.

Cyrek K, Socha P, Stefaniak K, Madeyska T, Mirosław-Grabowska J, Sudoł M, Czyżewski Ł (2010) Palaeolithic of Biśnik Cave (Southern Poland) within the environmental background. Quaternary International 220:5–30. https://doi.org/10.1016/j.quaint.2009.09.014

Daujeard C, Fernandes P, Guadelli J-L, Moncel M-H, Santagata C, Raynal J-P (2012) Neanderthal subsistence strategies in Southeastern France between the plains of the Rhone Valley and the mid-mountains of the Massif Central (MIS 7 to MIS 3). Quaternary International 252:32–47. https://doi.org/10.1016/j.quaint.2011.01.047

Daura J, Sanz M, Julià R, García-Fernández D, Fornós JJ, Vaquero M, Allué E, López-García JM, Blain HA, Ortiz JE, Torres T, Albert RM, Rodríguez-Cintas À, Sánchez-Marco A, Cerdeño E, Skinner AR, Asmeron Y, Polyak VJ, Garcés M, Arnold LJ, Demuro M, Pike AWG, Euba I, Rodríguez RF, Yagüe AS, Villaescusa L, Gómez S, Rubio A, Pedro M, Fullola JM, Zilhão J (2015) Cova del Rinoceront (Castelldefels, Barcelona): a terrestrial record for the Last Interglacial period (MIS 5) in the Mediterranean coast of the Iberian Peninsula. Quat Sci Rev 114:203–227. https://doi.org/10.1016/j.quascirev.2015.02.014

Del Grosso S, Parton W, Stohlgren T, Zheng D, Bachelet D, Prince S, Hibbard K, Olson R (2008) Global potential net primary production predicted from vegetation class, precipitation, and temperature. Ecology 89:2117–2126. https://doi.org/10.1890/07-0850.1

Demuro M, Arnold LJ, Aranburu A, Gómez-Olivencia A, Arsuaga J-L (2019) Single-grain OSL dating of the Middle Palaeolithic site of Galería de las Estatuas, Atapuerca (Burgos, Spain). Quaternary Geochronology 49:254–261. https://doi.org/10.1016/j.quageo.2018.02.006

Doerschner N, Fitzsimmons KE, Blasco R, Finlayson G, Rodríguez-Vidal J, Rosell J, Hublin J-J, Finlayson C (2019) Chronology of the Late Pleistocene archaeological sequence at Vanguard Cave, Gibraltar: Insights from quartz single and multiple grain luminescence dating. Quat Int 501: 289–302. [https://doi.org/10.1016/j.quaint.2018.02.02](https://doi.org/10.1016/j.quaint.2018.02.020)

Dublyansky Y, Shirokov V, Moseley GE, Kosintsev PA, Edwards RL, Spötl C (2021) ^230^ Th dating of flowstone from Ignatievskaya Cave, Russia: Age constraints of rock art and paleoclimate inferences. Geoarchaeology 36:532–545. <https://doi.org/10.1002/gea.21851>

Estévez J, Piqué R, Vila A, Taulé M, Weninger G, Bonet A, Clemente I (1993) El poblamiento prehistórico en la cuenca del Mediona (Alt Penedès, Barcelona). Trabalhos de Antropologia e Etnologia 33, 119–136

European Environment Agency (2023) Medium Resolution Net Primary Production (NPP, raster 196m) version 1, Nov. 2023

Fick SE, Hijmans RJ (2017) WorldClim 2: new 1-km spatial resolution climate surfaces for global land areas. International Journal of Climatology 37:4302–4315. <https://doi.org/10.1002/joc.5086>

Fiore I, Raynal JP, Tagliacozzo A (2005) Archeozoologia e aspetti tafonomici del sito musteriano di Baume Vallée (Massiccio centrale francese). In: Fiore I, Malerba G, Chilardi S (eds) Atti del 3° Convegno Nazionale di Archeozoologia, Siracusa, 3–5 novembre 2000. Studi di Paletnologia 2, Istituto Poligrafico e Zecca dello Stato, Roma, pp 81–92.

Foury Y, Desclaux E, Daujeard C, Defleur A, Moncel MH, Raynal JP (2016) Evolution of rodent populations in the middle Rhone Valley (Right Bank, Ardèche, France) during the late middle pleistocene and the early upper pleistocene from mis 6 to mis 4. Quaternaire 27: 55–79. <https://doi.org/10.4000/quaternaire.7527>

Frouin M, Douka K, Dave AK, Schwenninger J-L, Mercier N, Murray AS, Santaniello F, Boschian G, Grimaldi S, Higham T (2022) A refined chronology for the Middle and early Upper Paleolithic sequence of Riparo Mochi (Liguria, Italy). J Hum Evol 169: 103211. https://doi.org/10.1016/j.jhevol.2022.103211

Garcia J (2015) Long-term hominin occupation during the Middle and early Late Pleistocene: Chronostratigraphy of the Ter River basin (Girona, North-eastern Spain). Journal of Archaeological Science: Reports 3: 493–512. <https://doi.org/10.1016/j.jasrep.2015.07.005>

Goldberg P, Dibble H, Berna F, Sandgathe D, McPherron SJP, Turq A (2012) New evidence on Neandertal use of fire: Examples from Roc de Marsal and Pech de l’Azé IV. Quat Inter 247:325–340. https://doi.org/10.1016/j.quaint.2010.11.015

Grün R, Stringer C (2000) Tabun revisited: revised ESR chronology and new ESR and U-series analyses of dental material from Tabun C1. J Hum Evol 39:601–612. <https://doi.org/10.1006/jhev.2000.0443>

Grün R, Stringer C (2023) Direct dating of human fossils and the ever-changing story of human evolution. Quat Sci Rev 322:108379. <https://doi.org/10.1016/j.quascirev.2023.108379>

Guérin G, Frouin M, Talamo S, Aldeias V, Bruxelles L, Chiotti L, Dibble HL, Goldberg P, Hublin J-J, Jain M, Lahaye C, Madelaine S, Maureille B, McPherron SJP, Mercier N, Murray AS, Sandgathe D, Steele TE, Thomsen KJ, Turq A (2015) A multi-method luminescence dating of the Palaeolithic sequence of La Ferrassie based on new excavations adjacent to the La Ferrassie 1 and 2 skeletons. J Archaeol Sci 58: 147–166. https://doi.org/10.1016/j.jas.2015.01.019

Guérin G, Frouin M, Tuquoi J, Thomsen KJ, Goldberg P, Aldeias V, Lahaye C, Mercier N, Guibert P, Jain M, Sandgathe D, McPherron SJP, Turq A, Dibble HL (2017) The complementarity of luminescence dating methods illustrated on the Mousterian sequence of the Roc de Marsal: A series of reindeer-dominated, Quina Mousterian layers dated to MIS 3. Quat Int 433:102–115. https://doi.org/10.1016/j.quaint.2016.02.063

Hatton IA, McCann KS, Fryxell JM, Davies TJ, Smerlak M, Sinclair ARE, Loreau M (2015) The predator-prey power law: Biomass scaling across terrestrial and aquatic biomes. Science 349:aac6284. <https://doi.org/10.1126/science.aac6284>

Jacobs Z, Jankowski NR, Dibble HL, Goldberg P, McPherron SJP, Sandgathe D, Soressi M (2016) The age of three Middle Palaeolithic sites: Single-grain optically stimulated luminescence chronologies for Pech de l'Aze I, II and IV in France. J Hum Evol 95: 80–103. https://doi.org/10.1016/j.jhevol.2016.03.010

Jacobs Z, Li B, Shunkov MV, Kozlikin MB, Bolikhovskaya NS, Agadjanian AK, Uliyanov VA, Vasiliev SK, O’Gorman K, Derevianko AP, Roberts RG (2019) Timing of archaic hominin occupation of Denisova Cave in southern Siberia. Nature 565: 594–599. https://doi.org/10.1038/s41586-018-0843-2

Jaubert J (2002) Les occupations humaines contempo-raines du dernier interglaciaire et de la fin du stade 5dans le Sud de la France. In: Tuffreau A, Roebroeks W (eds) Le Dernier Interglaciaire et les occupations humaines du Paléolithique moyen. Université de Lille, pp 143-157.

Kandel AW, Sommer C, Kanaeva Z, Bolus M, Bruch AA, Groth C, Haidle MN, Hertler C, Heß J, Malina M, Märker M, Hochschild V, Mosbrugger V, Schrenk F, Conard NJ (2023) The ROCEEH Out of Africa Database (ROAD): A large-scale research database serves as an indispensable tool for human evolutionary studies. PLoS ONE 18:e0289513. https://doi.org/10.1371/journal.pone.0289513

Kaniewski D, Renault-Miskovsky J, de Lumley H (2005) Palaeovegetation from a Homo neanderthalensis occupation in Western Liguria: archaeopalynology of Madonna dell’Arma (San Remo, Italy). J Archaeol Sci 32: 827–840. https://doi.org/10.1016/j.jas.2004.12.005

Karavanić I, Hellstrom J, Rabeder G, Vukosavljević N, Banda M, Smith FH (2021) New U-Th dates from Vindija, Velika pećina (Kličevica) and Mujina pećina and their implications for chronology of the Middle Paleolithic in Croatia. Coll Antropol 45(1): 1–10.

Kehl M, Eckmeier E, Franz SO, Lehmkuhl F, Soler J, Soler N, Reicherter K, Weniger G-C (2014) Sediment sequence and site formation processes at the Arbreda Cave, NE Iberian Peninsula, and implications on human occupation and climate change during the Last Glacial. Climate of the Past 10:1673–1692. https://doi.org/10.5194/cp-10-1673-2014

Koehler H, Wegmüller F, Audiard B, Auguste P, Bahain J-J, Bocherens H, Diemer S, Preusser F, Pümpin C, Sévêque N, Stoetzel E, Tombret O, Wuscher P (2021) The Middle Paleolithic Occupations of Mutzig-Rain (Alsace, France). https://doi.org/10.5167/UZH-213104

Kosintsev PA, Bachura OP (2013) Late Pleistocene and Holocene mammal fauna of the Southern Urals. Quaternary International 284:161–170. https://doi.org/10.1016/j.quaint.2012.06.022

Laplana C, Herráez E, Yravedra Saínz De Los Terreros J, Bárez S, Rubio-Jara S, Panera J, Rus I, Pérez-González A (2015) Biocronología de la Terraza Compleja de Butarque del río Manzanares en el Estanque de Tormentas al sur de Madrid (España). Estud geol 71:e028. https://doi.org/10.3989/egeol.41808.338

Laurat T, Brühl E (2021) Neumark-Nord 2 – A multiphase Middle Palaeolithic open-air site in the Geisel Valley (Central Germany). L’Anthropologie 125:102936. https://doi.org/10.1016/j.anthro.2021.102936

Lieth H (1975) Modeling the Primary Productivity of the World. In: Lieth H, Whittaker RH (eds) Primary Productivity of the Biosphere. Springer, Berlin, Heidelberg, pp 237–263

Lopez-García JM, Berto C, Peresani M (2019) Environmental and climatic context of the hominin occurrence in northeastern Italy from the late Middle to Late Pleistocene inferred from small-mammal assemblages. Quat Sci Rev 216: 18–33. <https://doi.org/10.1016/j.quascirev.2019.05.025>

López-Recio M, Baena J, Silva PG (2018) La secuencia cronocultural de la ocupación paleolítica en el valle inferior del río Manzanares (Madrid, España). Cuat. Geomorfol. 32(3-4): 57–88. https://doi.org/10.17735/cyg.v32i3-4.64210

Marín J, Daujeard C, Saladié P, Rodríguez-Hidalgo A, Vettese D, Rivals F, Boulbes N, Crégut-Bonnoure E, Lateur N, Gallotti R, Arbez L, Puaud S, Moncel M-H (2020) Neanderthal faunal exploitation and settlement dynamics at the Abri du Maras, level 5 (south-eastern France). Quat Sci Rev 243:106472. <https://doi.org/10.1016/j.quascirev.2020.106472>

Martín-Perea DM, Maíllo-Fernández JM, Marín J, Arroyo X, Asiaín R (2023) A step back to move forward: a geological re-evaluation of the El Castillo Cave Middle Palaeolithic lithostratigraphic units (Cantabria, northern Iberia). J. Quat. Sci. 38(2): 221–234. <https://doi.org/10.1002/jqs.3473>

Mercier N, Martin L, Kreutzer S, Moineau V, Cliquet D (2019) Dating the palaeolithic footprints of ‘Le Rozel’ (Normandy, France). Quat Geochronol 49:271–277. https://doi.org/10.1016/j.quageo.2017.12.005

Mihailović D, Milošević S, Blackwell BAB, Mercier N, Mentzer SM, Miller CE, Morley MW, Bogićević K, Đurić D, Marković J, Mihailović B, Dragosavac S, Plavšić S, Skinner AR, Chaity IIC, Huang YEW, Chu S, Nenadić D, Radović P, Lindal J, Roksandic M (2022) Neanderthal settlement of the Central Balkans during MIS 5: Evidence from Pešturina Cave, Serbia. Quaternary International 610:1–19. <https://doi.org/10.1016/j.quaint.2021.09.003>

Moncel M-H, Lhomme G (2007) Les assemblages lithiques des niveaux du Paléolithique moyen de l’Abri des Pêcheurs (Ardèche, sud-est de la France). Des occupations néandertaliennes récurrentes dans un fossé. L’Anthropologie 111: 211–253.

Moncel M-H, Michel V (2000) Première datation radiométrique par U-Th d’un niveau moustérien de l’Abri du Maras (Ardèche, France). Bulletin de la Société préhistorique française 97:371–375

Moreno D, Ortega AI, Falguères C, Shao Q, Tombret O, Gómez-Olivencia A, Aranburu A, Trompier F, Bermúdez De Castro JM, Carbonell E, Arsuaga JL (2022) ESR/U-series chronology of the Neanderthal occupation layers at Galería de las Estatuas (Sierra de Atapuerca, Spain). Quat Geochronol 72:101342. https://doi.org/10.1016/j.quageo.2022.101342

Nejman L, Rhodes E, Škrdla P, Tostevin G, Neruda P, Nerudová Z, Valoch K, Oliva M, Kaminská L, Svoboda JA, Grün R (2011) New Chronological Evidence for the Middle to Upper Palaeolithic Transition in the Czech Republic and Slovakia: New Optically Stimulated Luminescence Dating Results. Archaeometry 53:1044–1066. https://doi.org/10.1111/j.1475-4754.2011.00586.x

Niven L, Steele TE, Rendu W, Mallye J-B, McPherron SP, Soressi M, Jaubert J, Hublin J-J (2012) Neandertal mobility and large-game hunting: The exploitation of reindeer during the Quina Mousterian at Chez-Pinaud Jonzac (Charente-Maritime, France). Journal of Human Evolution 63:624–635. https://doi.org/10.1016/j.jhevol.2012.07.002

Patou-Mathis M, Chabaï V (2003) Kabazi II (Crimée, Ukraine): un site d’abattage et de boucherie du Paléolithique moyen. L’Anthropologie 107:223–253. https://doi.org/10.1016/S0003-5521(02)00006-7

Puzachenko AYu, Titov VV, Kosintsev PA (2021) Evolution of the European regional large mammals assemblages in the end of the Middle Pleistocene – The first half of the Late Pleistocene (MIS 6–MIS 4). Quaternary International 605–606:155–191. <https://doi.org/10.1016/j.quaint.2020.08.038>

Raynal JP, Decroix C (1986) L’abri de Baume-Vallée (Haute-Loire, France), site moustérien de moyenne montagne dans son contexte régional. Arqueologia, Porto 15:17–42.

Raynal JP, Le Corre-Le Beux M, Santagata C, Fernandes P, Guadelli JL, Fiore I, Tagliacozzo A, Lemorini C, Rhodes EJ, Bertran P, Kieffer G, Vivent D (2005) Paléolithique moyen dans le Sud du Massif central: les données du Velay (Haute-Loire, France). In: Molines N, Moncel MH, Monnier JL (eds) Les premiers peuplements en Europe. British Archaeological Reports International Series S1364, Oxford, pp 173–202.

Richard M, Pons-Branchu E, Genuite K, Jaillet S, Joannes-Boyau R, Wang N, Genty D, Cheng H, Price GJ, Pierre M, Dapoigny A, Falguères C, Tombret O, Voinchet P, Bahain J-J, Moncel M-H (2021) Timing of Neanderthal occupations in the southeastern margins of the Massif Central (France): A multi-method approach. Quat Sci Rev 273:107241. https://doi.org/10.1016/j.quascirev.2021.107241

Richter D, Hublin J-J, Jaubert J, McPherron SP, Soressi M, Texier J-P (2013) Thermoluminescence dates for the Middle Palaeolithic site of Chez-Pinaud Jonzac (France). Journal of Archaeological Science 40:1176–1185. https://doi.org/10.1016/j.jas.2012.09.003

Richter D, Mercier N, Valladas H, Jaubert J, Texier P-J, Brugal J-P, Kervazo B, Reyss J-L, Joron J-L, Wagner GA (2007) Thermoluminescence dating of heated flint from the Mousterian site of Bérigoule, Murs, Vaucluse, France. Journal of Archaeological Science 34:532–539. https://doi.org/10.1016/j.jas.2006.06.006

Rink WJ, Grün R, Yalcinkaya E, Otte M, Taşkiran H, Valladas H, Mercier N, Bar-Yosef O, Kozlowski JK, Schwarcz HP (1994) ESR Dating of the Last Interglacial Mousterian at Karaïn Cave, Southern Turkey. Journal of Archaeological Science 21:839–849. https://doi.org/10.1006/jasc.1994.1081

Rink WJ, Kandel AW, Conard NJ (2002) The ESR geochronology and geology of the open-air Palaeolithic deposits in Bollschweil, Germany. Archaeometry 44:635–650. https://doi.org/10.1111/1475-4754.t01-1-00090

Rink WJ, Schwarcz HP, Smith FH, Radovĉiĉ J (1995) ESR ages for Krapina hominids. Nature 378:24–24. https://doi.org/10.1038/378024a0

Roditi E, Starkovich BM (2022) Investigating Middle Palaeolithic subsistence: zooarchaeological perspectives on the potential character of hominin climate refugia in Greece. Journal of Quaternary Science 37:181–193. https://doi.org/10.1002/jqs.3371

Rodríguez J, Blain HA, Mateos A, Martín-González JA, Cuenca-Bescós G, Rodríguez-Gómez G (2014) Ungulate carrying capacity in Pleistocene Mediterranean ecosystems: Evidence from the Atapuerca sites. Palaeogeography, Palaeoclimatology, Palaeoecology 393:122–134. <https://doi.org/10.1016/j.palaeo.2013.11.011>

Rodríguez-Álvarez XP, de Lombera Hermida A, Fábregas-Valcarce R, Lazuén Fernández T (2011) The Upper Pleistocene site of Cova Eirós (Triacastela, Lugo, Spain). In: de Lombera Hermida A, Fábregas-Valcarce R (eds) To the West of Spanish Cantabria: The Palaeolithic Settlement of Galicia. BAR International Series 2283, Oxford, 123–133.

Rouzaud F, Soulier M, Brugal J-P, Jaubert J (1990) L’Igue des Rameaux (Saint-Antonin-Noble-Val, Tarn-et-Garonne). Un nouveau gisement du Pléistocène moyen : premiers résultats. PALEO : Revue d’Archéologie Préhistorique 2:89

Rufà A, Blasco R, Roger T, Rué M, Daujeard C (2018) A rallying point for different predators: the avian record from a Late Pleistocene sequence of Grotte des Barasses II (Balazuc, Ardèche, France). Archaeol Anthropol Sci 10:1459–1476. <https://doi.org/10.1007/s12520-017-0469-6>

Rusch L, Grégoire S, Pois V, Moigne A-M (2019) Neanderthal and carnivore occupations in unit II from the Upper Pleistocene site of Ramandils Cave (Port-la-Nouvelle, Aude, France). J Archaeol Sci Rep 28: 102038. https://doi.org/10.1016/j.jasrep.2019.102038

Sabol M, Slyšková D, Bodoriková S, Čejka T, Čerňanský A, Ivanov M, Joniak P, Kováčová M, Tóth C (2017) Revised floral and faunal assemblages from Late Pleistocene deposits of the Gánovce-Hrádok Neanderthal site – biostratigraphic and palaeoecological implications. FI 73:182–196. <https://doi.org/10.2478/if-2017-0010>

Sánchez Yustos P, Díez Martín F, Díaz Muñoz IM, Gómez de la Rúa D, Gómez González JA (2011) Estrategias de talla en Cueva Corazón (Mave, Palencia). Un yacimiento del Musteriense Antiguo en las estribaciones meridionales de la Cordillera Cantábrica. Trabajos de Prehistoria 68(1): 51–63. https://doi.org/10.3989/tp.2011.11058

Sesé C, López-Martínez N (2013) Nuevos datos paleontológicos del Pleistoceno en el Valle del Manzanares (Madrid, España): Los micromamíferos del yacimiento del Arenero de Arriaga. New paleontological data from the Pleistocene Manzanares Valley (Madrid, Spain): The micromammals from the Arenero de Arriaga site. https://doi.org/10.3989/egeol.41318.270

Sesé C, Soto E (2005) Mamíferos del yacimiento del Pleistoceno Medio de Ambrona (Soria, España): Análisis faunístico e interpretación paleoambiental. Madrid (Comunidad Autónoma)

Silva PG, López-Recio M, Tapias F, Roquero E, Morín J, Rus I, Carrasco-García P, Giner-Robles JL, Rodríguez-Pascua MA, Pérez-López R (2013) Stratigraphy of the Arriaga Palaeolithic sites. Implications for the geomorphological evolution recorded by thickened fluvial sequences within the Manzanares River valley (Madrid Neogene Basin, Central Spain). Geomorphology 196:138–161. https://doi.org/10.1016/j.geomorph.2012.10.019

Stiner MC (1993) Small Animal Exploitation and its Relation to Hunting, Scavenging, and Gathering in the Italian Mousterian. Archaeological Papers of the American 4:107–125. https://doi.org/10.1525/ap3a.1993.4.1.107

Stoetzel E, Koehler H, Cliquet D, Sévêque N, Auguste P (2016) New data on Late Pleistocene small vertebrates from northern France. C R Palevol 15:681–695. <https://doi.org/10.1016/j.crpv.2015.12.003>

Suárez-Bilbao A, Garcia-Ibaibarriaga N, Castaños J, Castaños P, Iriarte-Chiapusso MJ, Arrizabalaga A, Torres T, Ortiz JE, Murelaga X (2016) A new Late Pleistocene non-anthropogenic vertebrate assemblage from the northern Iberian Peninsula: Artazu VII (Arrasate, Basque Country). C R Palevol 15:950–957. <https://doi.org/10.1016/j.crpv.2016.05.002>

Torres, T., Ortiz, J.E., Fernández, E., Arroyo-Pardo, E., Grün, R., Pérez-González, A., 2014. Aspartic acid racemization as a dating tool for dentine: A reality. Quaternary Geochronology 22, 43–56. https://doi.org/10.1016/j.quageo.2014.02.004

Vaissié E, Delvigne V, Faivre JP, Fernandes P, Turq A, Raynal JP (2017) Techno-économie et signification culturelle de l’occupation moustérienne supérieure de Baume-Vallée (Haute-Loire). Comptes Rendus Palevol 16:804–819. <https://doi.org/10.1016/j.crpv.2017.06.005>

Valensi P, Psathi E (2004) Faunal Exploitation during the Middle Palaeolithic in south-eastern France and north-western Italy. International Journal of Osteoarchaeology 14:256–272. <https://doi.org/10.1002/oa.760>

Valladas H, Mercier N, Falguères C, Bahain JJ (1999) Contribution des méthodes nucléaires à la chronologie des cultures paléolithiques entre 300 000 et 35 000 ans BP. Gallia Préhistoire 41: 153–166. https://doi.org/10.3406/galip.1999.2163

Vermeersch PM (2025) Radiocarbon Palaeolithic Europe Database, Version 31

Vidal-Cordasco M, Ocio D, Hickler T, Marín-Arroyo AB (2022) Ecosystem productivity affected the spatiotemporal disappearance of Neanderthals in Iberia. Nat Ecol Evol 6:1644–1657. https://doi.org/10.1038/s41559-022-01861-5

Villa P, Soriano S, Pollarolo L, Smriglio C, Gaeta M, D’Orazio M, Conforti J, Tozzi C (2020) Neandertals on the beach: Use of marine resources at Grotta dei Moscerini (Latium, Italy). PLoS ONE 15:e0226690. <https://doi.org/10.1371/journal.pone.0226690>

Vonhof HB, Verheyden S, Bonjean D, Pirson S, Weber M, Scholz D, Hellstrom J, Cheng H, Jia X, Di Modica K, Abrams G, van Nunen MAP, Ruiter J, van der Does M, Böhl D, van der Lubbe JHJL (2024) Improving the age constraints on the archeological record in Scladina Cave (Belgium): new speleothem U–Th ages. Clim Past 20: 2741–2758. https://doi.org/10.5194/cp-20-2741-2024

Willmes M, Grün R, Douka K, Michel V, Armstrong RA, Benson A, Crégut-Bonnoure E, Desclaux E, Fang F, Kinsley L (2016) A comprehensive chronology of the Neanderthal site Moula-Guercy, Ardèche, France. J Archaeol Sci Report 9:309–319. <http://dx.doi.org/10.1016/j.jasrep.2016.08.003>

Wiśniewski A, Bobak D, Połtowicz-Bobak M, Moska P (2024) Late Middle Palaeolithic and Early Upper Palaeolithic in Poland in the light of new numerical dating. Geogr. Pol. 97(3): 295–325. https://doi.org/10.7163/GPol.0281

Wojtal P, Patou-Mathis M (2003) Subsistence behaviours in a Middle Palaeolithic site in Poland: the Raj Cave. Br Archaeol Rep Int Ser 1105:83–89

Yokoyama Y, Nguyen H-V, Quaegebeur J-P, Hasif GL (1987) Datation par la spectrométrie gamma non destructive et la resonance de spin électronique (ESR) du remplissage de la grotte de 1’Arbreda. 137–143

Zilhão J, Angelucci DE, Arnold LJ, Demuro M, Hoffmann DL, Pike AWG (2021) A revised, Last Interglacial chronology for the Middle Palaeolithic sequence of Gruta da Oliveira (Almonda karst system, Torres Novas, Portugal). Quat Sci Rev 258: 106885. https://doi.org/10.1016/j.quascirev.2021.106885

Zilhão J, Angelucci DE, Igreja MA, Arnold LJ, Badal E, Callapez P, Cardoso JL, d’Errico F, Daura J, Demuro M, Deschamps M, Dupont C, Gabriel S, Hoffmann DL, Legoinha P, Matias H, Monge Soares AM, Nabais M, Portela P, Queffelec A, Rodrigues F, Souto P (2020) Last Interglacial Iberian Neandertals as fisher-hunter-gatherers. Science 367:eaaz7943. https://doi.org/10.1126/science.aaz7943

Zilhão J, Cardoso J, Pike A, Weninger B (2011) Gruta Nova da Columbeira (Bombarral, Portugal): Site stratigraphy, age of the Mousterian sequence, and implications for the timing of Neanderthal extinction in Iberia. Quartär 58:93–112
